# Enhanced Oxygen Redox Activity and Structure Stability of P2‐Type Manganese‐Based Cathodes Through Medium‐Entropy Strategy

**DOI:** 10.1002/advs.202518795

**Published:** 2026-01-04

**Authors:** Dongxiao Wang, Yuxuan Liu, Zihao Wang, Wei Su, Xingguo Qi, Huican Mao, Kan Zhang, Shigang Lu, Bingkun Guo, Yingchun Lyu

**Affiliations:** ^1^ Materials Genome Institute & State Key Laboratory of Materials for Advanced Nuclear Energy Shanghai University Shanghai China; ^2^ HiNa Battery Technology Co., Ltd Liyang China; ^3^ Department of Materials Science and Key Laboratory of Automobile Materials MOE, Jilin University Changchun China; ^4^ College of Sciences and Institute for Sustainable Energy Shanghai University Shanghai China

**Keywords:** anionic redox, ionic potential, medium‐entropy, P2‐type cathodes, sodium‐ion battery

## Abstract

Oxygen redox reaction offers a promising strategy to enhance the energy density of manganese‐based layered transition‐metal oxides, yet the associated multiple structural transitions and volume changes usually undermine long‐term stability. Entropy stabilization, which leverages elemental synergy to improve structural robustness, has emerged as a promising solution. Here, we integrate ionic potential into medium‐entropy design to guide element selection. Taking Na_0.67_Ni_0.33_Mn_0.67_O_2_ as an example, doping with multiple low ionic potential elements elevates O 2p degeneracy, thereby enhancing and stabilizing high voltage oxygen redox reactions. A medium‐entropy P2‐type oxide, Na_0.8_Li_0.1_Ni_0.1_Cu_0.1_Ti_0.1_Mn_0.6_O_2_, demonstrates a high reversible capacity of 223.7 mAh g^−1^ and an energy density of 616.3 Wh kg^−1^, while maintaining 87% capacity retention over 200 cycles. It markedly suppresses transition metal layer gliding and Jahn–Teller distortion, stabilizes Mn^3+^ against disproportionation to preserve Mn redox activity and suppress voltage decay, while detrimental phase transitions are fully inhibited. This strategy simultaneously boosts reversible capacity and leverages entropy‐driven phase stabilization, offering a practical route toward next‐generation, high‐capacity, durable sodium‐ion batteries.

## Graphical abstract

A medium‐entropy strategy guided by ionic potential is applied to weaken TM─O bonds and suppress Jahn–Teller distortion in P2‐type cathodes, thereby enhancing the oxygen redox activity and structure stability. A medium‐entropy oxide, Na_0.8_Li_0.1_Ni_0.1_Cu_0.1_Ti_0.1_Mn_0.6_O_2_, demonstrates a high reversible capacity of 223.7 mAh g^−1^ and an energy density of 616.3 Wh kg^−1^, while maintaining 87% capacity retention over 200 cycles.

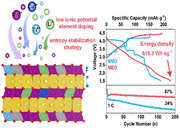

## 1 Introduction

Manganese‐based layered transition metal (TM) oxides and derivatives are promising for large‐scale applications due to their low cost. Among them, P2‐type structures offer superior rate capability, cycling life, and energy density [1, 2]. This is because of their open Na^+^ diffusion pathways, wide interlayer spacings, and high operating voltages. However, these materials suffer from structural instability during cycling. At high voltages during desodiation, increased Coulombic repulsion triggers a detrimental P2 to O2 phase transition [3, 4]. Conversely, at low voltages during sodiation, the reduction of Mn^4+^ to high‐spin Mn^3+^ (t_2g_^3^‐e_g_^1^), induces Jahn‐Teller distortion. This leads to anisotropic volume expansion and a transition to a P′2 phase. Both phase transitions cause significant damage due to the large differences in crystallographic parameters, resulting in stress accumulation, structural degradation, and eventual collapse [4, 5, 6]. Furthermore, oxygen redox reactions contribute to performance decay. These reactions, involving changes in the oxidation state of oxygen, can cause lattice oxygen release and TM ion migration. This manifests as severe voltage decay and a short cycle life [7].

Local structure regulation and element doping are common methods for suppressing the phase transitions and enhancing the cycling stability [8, 9]. Reducing the Na content and increasing the ratio of NaO_2_ slab spacing to TMO_2_ slab spacing (R = d_O−Na−O_/d_O−TM−O_) helps achieve highly reversible phase transitions and inhibit cation migration [10]. Introducing high ionic potential elements into the TM layer can construct robust bonds with oxygen to localize the electrons and improve the stability of the local structure [11]. However, lowering the Na content may raise the valence of TM layer elements and weaken cation redox, while enhanced TM─O bonds concurrently suppress oxygen redox and diminish anion capacity. An emerging solution is the entropy stabilization strategy, which uses multi‐element doping to create local disorder and enhance structural stability [12, 13, 14]. While high‐entropy oxides can achieve remarkable stability, they often require a high content of non‐active elements, which sacrifices reversible capacity. In particular, reducing configurational entropy from ultrahigh to moderate levels to balance entropy increase and capacity utilization in medium‐entropy oxides (MEO), severe lattice distortion or strain may occur due to the prominent mismatches in ionic mass, size, and bond state [15, 16]. Therefore, developing a design strategy to enhance reversible capacity while maximizing entropy dominated phase stabilization is crucial for advancing the development of outstanding layered cathode materials.

By integrating ionic potential into battery design, researchers can predict and optimize electrode/electrolyte crystal structures for efficient ion transport and stability, while also forecasting material properties during cycling to clarify mechanisms and guide performance optimization [17, 18]. The activity of oxygen redox is primarily constrained by the covalent strength of the TM─O bond [19, 20]. Therefore, doping with transition metals of low ionic potential is expected to weaken the TM─O bond, thereby significantly enhancing the additional capacity derived from oxygen redox reactions. Furthermore, calculations based on the cationic potential formula and phase maps indicate that reducing the ionic potential of transition metals can increase the sodium content without altering the material's structure. Building on this rationale and inspired by the entropy stabilization paradigm, this study synthesized a medium‐entropy P2‐type layered oxide, Na_0.8_Li^+^_0.1_Ni^2+^_0.1_Cu^2+^_0.1_Mn^3+^_0.1_Mn^4+^_0.5_Ti^4+^_0.1_O_2_, with high sodium content through the co‐doping of multiple low ionic potential elements. Among these, Li^+^, Cu^2+^, Mn^3+^, and Ti^4+^ possess some of the lowest ionic potentials for their respective valence states among elements commonly used in sodium‐ion cathode materials.

The doping of these multiple low ionic potential elements increases the degeneracy of the O 2p states, which enhances both the activity and stability of high voltage oxygen redox reactions. Concurrently, it effectively suppresses phase transitions at low voltages. Moreover, this strategy improves ionic transport kinetics and overall cycling stability. This comprehensive optimization addresses multiple challenges faced by conventional battery materials, paving the way for the development of next‐generation energy storage solutions that are both high‐performing and durable.

## 2 Results and Discussion

### 2.1 Crystal Structure Characterization

**FIGURE 1:**
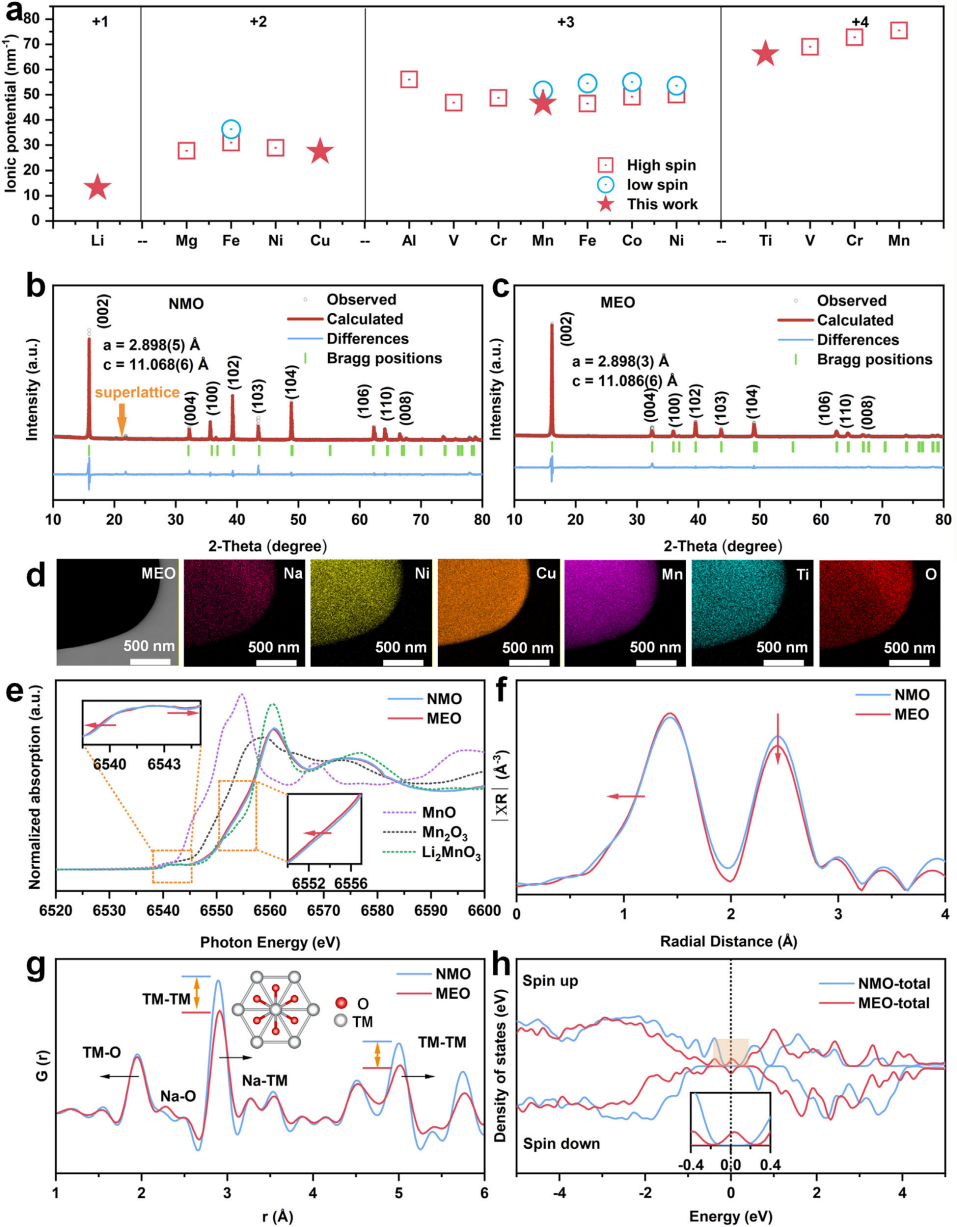
Crystal and local structure. (a) Ionic potentials of elements in sodium ion layered oxides, these elements are classified based on their oxidation states and relative atomic masses. Rietveld refinements of XRD of (b) NMO and (c) MEO compounds. (d) EDS mappings of MEO. (e) The Mn *K‐edge* XANES spectra for the NMO and MEO samples with reference spectra of MnO, Mn_2_O_3_ and Li_2_MnO_3_. (f) Mn *K‐edge* FT‐EXAFS spectra of NMO and MEO samples. (g) X‐ray PDF of MEO and NMO. (h) Calculated total density of states (TDOS) of MEO and NMO.

3d TM and some main group ions that can occupy the TM sites were selected [17]; while 4d and other expensive elements were avoided. Figure 1a shows the ionic potential of various candidate elements. The ions with the lowest ionic potential in each valence state (Li^+^, Cu^2+^, Mn^3+,^ and Ti^4+^) were selected to construct a medium‐entropy configuration. Finally, the target composition, Na_0.8_Li^+^_0.1_Ni^2+^_0.1_Cu^2+^_0.1_Mn^3+^_0.1_Mn^4+^_0.5_Ti^4+^_0.1_O_2_, shows a lower ionic potential and a higher R factor of 55.95 nm^−1^ and 1.23, compared to those of Na_0.67_Ni_0.33_Mn_0.67_O_2_ (60.13 nm^−1^ and 0.67). Computational details are provided in Figure S1 and Table S1. Both materials were synthesized via a solid‐state reaction and are hereafter referred to as NMO and MEO, respectively. The inductively coupled plasma (ICP) results (Table S2) confirmed that the actual compositions of the samples align with theoretical compositions. Although the cationic potential of MEO sample lies in the biphasic zone in the cationic potential phase map (Figure S2), it exhibits a pure P2 structure in a wide synthesis temperature range of 750°C–950°C (Figure S3). This deviation underscores the dominant role of configurational entropy in stabilizing the single‐phase solid solution, as the high entropy of mixing lowers the Gibbs free energy (ΔG = ΔH−TΔS) [21, 22, 23]. Figure 1b,c presents the X‐ray diffraction (XRD) patterns and Rietveld refinement results for MEO and NMO, with detailed crystallographic data in Tables S3 and S4. Both samples crystallize in a P2‐type layered structure. Notably, MEO exhibits an enlarged slab spacing in the NaO_2_ layer (3.859 Å) and a compressed inters lab spacing in the TM layer (2.375 Å) compared to NMO (3.425 and 2.693 Å, respectively). The expanded NaO_2_ spacing enhances Na^+^ diffusion, while the compressed TM layer strengthens the TM─O bonds, collectively hindering cation migration and mitigating transient lattice parameter fluctuations during cycling, thereby inhibiting cracking and structural degradation [10]. The absence of the superlattice peaks at 20° and 22° in MEO sample suggests the suppression of transition metal ordering [24]. The introduced cations with diverse charge states and ionic radii create a disordered local environment [25], which further disrupts the coupled charge ordering and Na^+^/vacancy order [26]. This Na^+^/vacancy disorder would enhance electrode kinetics and improve ionic diffusion efficiency of the sample [26]. Scanning electron microscopy (SEM) images (Figure S4) show well‐crystallized particles of 2–3 µm for both samples. STEM‐EDX mappings (Figure 1d; Figure S5) confirm a uniform elemental distribution in NMO and MEO. It further confirms the single phase in MEO.

Figure 1e displays the normalized Mn *K‐edge* XANES spectra of NMO and MEO. Relative to NMO, the absorption edge of MEO shifts to lower energy. Integral analysis yields average Mn valences of +4.00 for NMO and +3.84 for MEO, which are not significantly different from the theoretical compositions (see Figure S6 for details). The pre‐edge peak corresponds to the 1s→3d transition [27]; the inset in Figure 1e shows that this peak is significantly broadened in MEO, a consequence of local distortions around Mn centers. This enhanced disorder can improve the mechanical and chemical stability of materials during the (de)sodiation process.[28]. These distortions are further corroborated by the corresponding EXAFS spectra (Figure 1f), the Mn─TM peaks of MEO display more significant attenuation, confirming that multi‐element doping induces local structural disorder around Mn [29]. Figure S7 shows the normalized Ni *K‐edge* XANES spectra of NMO and MEO. The Ni ions in both samples exhibit the same +2 oxidation state, and the Ni EXAFS spectra of MEO show the same local structural distortions as those of Mn (Figure S8). The local TM ordering in MEO and NMO was further examined by X‐ray pair distribution function (PDF) analysis. In the short‐range structure (1–6 Å) (Figure 1g), the TM─O bond in MEO shifts toward the low‐r direction, while the TM─TM bond shifts toward the higher‐r region compared with NMO, indicating a reduced TMO_2_ slab spacing that is in line with the XRD results. In addition, the full‐width at half‐maximum (FWHM) of the TM─TM peak is markedly broadened, consistent with the local structural distortion around TM revealed by XAS analysis [30]. In the long‐range structure (6–20 Å) (Figure S9) indicates that MEO maintains a similar long‐range order with NMO. The electronic structures of the samples were evaluated using the total density of states (TDOS) obtained from DFT calculations (Figure 1h; Figure S10). The TDOS of MEO exhibits a finite density of states at the Fermi level (*E*_F_), indicating metallic behavior, in sharp contrast to the gapped insulator NMO. This metallic character arises from the partial occupation of Mn 3d states due to the increased Mn^3+^/Mn^4+^ mixed valence (average Mn oxidation state ≈+3.83), which collapses the Mott–Hubbard gap [31, 32]. Projected DOS (PDOS) analysis (Figure S11) was employed to deconvolute the orbital contributions. It confirms that the states at *E*_F_ are dominated by Mn *e*_g_^*^ orbitals, with a minor O 2p contribution (about 25% of the Mn peak height), signifying Mn─O hybridized metallic states with prevailing Mn *e*_g_^*^ character. The enhanced electronic conductivity of MEO consequently contributes to its improved electrochemical kinetics [33, 34].

### 2.2 Electrochemical Characterization

**FIGURE 2:**
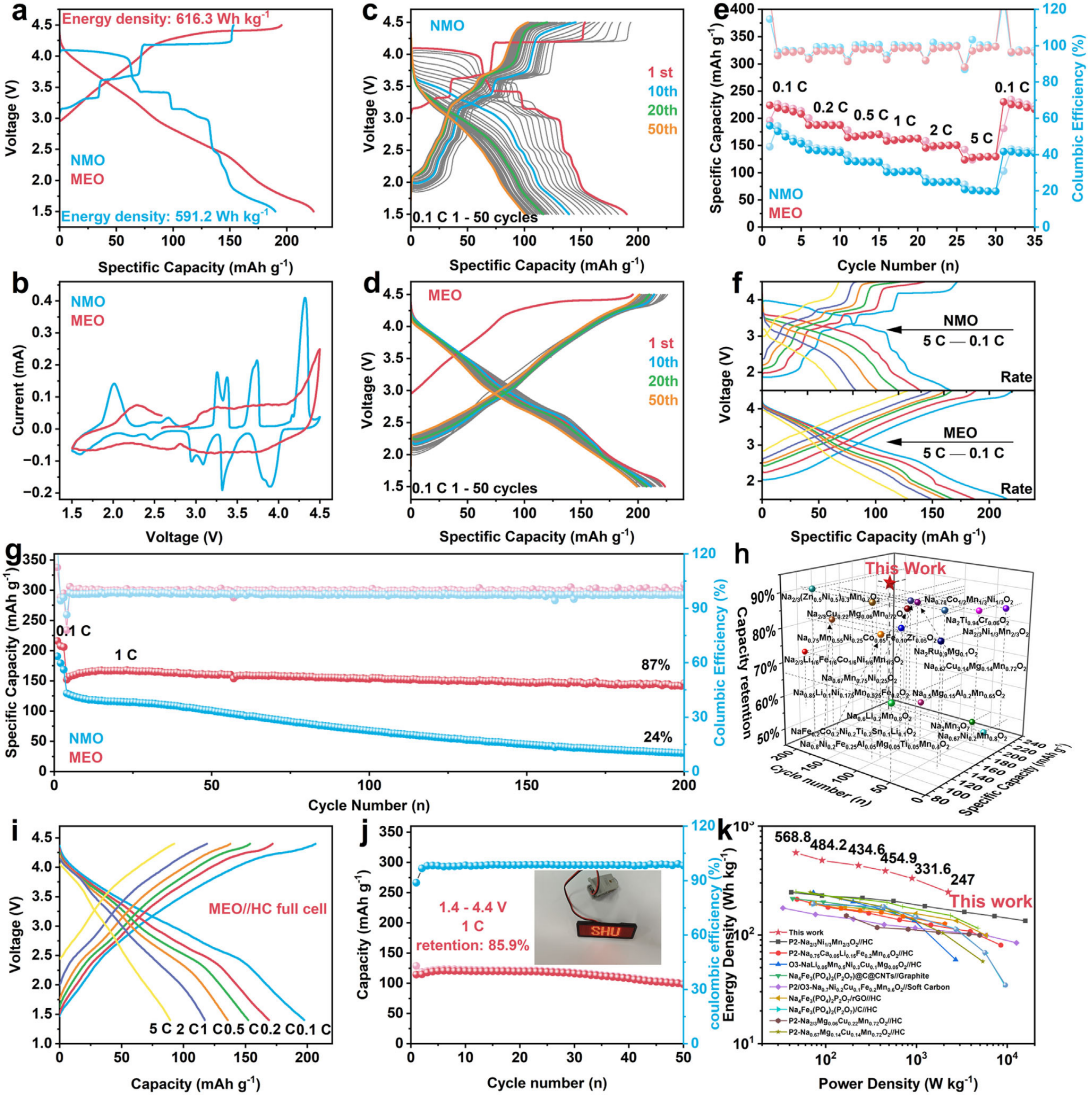
Electrochemical performance. (a) The first charge‐discharge voltage profiles of NMO and MEO samples. (b) Cyclic voltammograms of the NMO and MEO electrodes. The charge/discharge profiles for the first 50 cycles of (c) NMO and (d) MEO. (e) Rate capability of NMO and MEO cathodes. (f) Charge–discharge curves of NMO and MEO at different rates. (g) Cycling performance of NMO and MEO samples. (h) Statistics of charge/discharge capacity and cycle stability for some representative SIB cathode materials. (i) Charge–discharge curves of MEO//HC full cell at different rates. (j) Cycling performance at 1 C with an inset of the lighting sign‐ ‘SHU’ for the full cells. (k) Ragone plot of MEO//HC full cell and comparison with recently reported works.

To assess the impact of low ionic potential medium‐entropy design on Na^+^ storage performance, coin‐type half cells were constructed. Figure 2a shows the charge/discharge profiles of NMO and MEO materials at 0.1 C (1 C = 200 mA g^−1^) within 1.5–4.5 V. The NMO electrode exhibits multiple distinct voltage plateaus, corresponding to the sequential rearrangement of Na^+^/vacancy ordering. In contrast, the MEO electrode shows an effectively smooth the charge–discharge profiles, in agreement with the loss of Na^+^/vacancy ordering discussed before. The MEO electrode demonstrates higher charge (195.5 mAh g^−1^) and discharge (223.7 mAh g^−1^) capacities than that of NMO (153.3 and 190 mAh g^−1^). Additionally, MEO achieves a discharge energy density of 616.3 Wh kg^−1^, surpassing that of NMO (591.2 Wh kg^−1^). It confirms that low ionic potential doping effectively enhances both capacity and energy density. Figure 2b shows the cyclic voltammetry (CV) profiles. The NMO electrode displays sharp redox peaks between 2.0 and 4.0 V, corresponding to the Ni^2+^/Ni^3+^ redox couple. The significant peak splitting belongs to the process involving Na⁺/vacancy ordering [35]. The anodic peaks above 4.2 V correspond to oxygen activity, while those below 2.0 V arise from the Mn^3+^/Mn^4+^ redox couple [36]. In contrast, CV curves of MEO electrode are notably smoother than that of NMO, confirming that the medium‐entropy design effectively suppresses unfavorable Na^+^/vacancy ordering. Figure 2c,d shows the galvanostatic charge/discharge profiles of NMO and MEO cathodes cycled at 0.1 C. Over the first 50 cycles, the voltage plateau of NMO rapidly transforms into a sloped curve, indicating irreversible crystal structure changes and damage [37]. Consequently, the capacity of NMO significantly decreases to 100 mAh g^−1^, accompanied by severe voltage decay. In contrast, the MEO maintains a specific capacity of 200 mAh g^−1^ and only a slight voltage decay after 50 cycles, demonstrating that medium‐entropy doping effectively enhances cycling stability. As illustrated in Figure 2e, MEO sample demonstrated exceptional rate performance. At a 5 C rate, it maintained a specific capacity of 129.6 mAh g^−1^, while NMO only achieved a specific capacity of 65.7 mAh g^−1^. Figure 2f presents the charge/discharge curves of NMO and MEO electrodes across various rates. Notably, the MEO electrode exhibits consistent curve patterns regardless of the rate, while the high‐voltage plateaus of the NMO electrode significantly diminish at higher currents. This behavior indicates that the MEO electrode undergoes considerably lower electrochemical polarization and sustains rapid transport kinetics. As a result, MEO can swiftly adapt to current changes, enabling it to preserve both a high specific capacity and a stable voltage profile even at elevated rates, with additional details provided in Figure S12 [38]. Figure 2g clearly shows that the MEO sample has excellent cycle stability. After 200 cycles, it keeps a specific capacity of 146.3 mAh g^−1^, retaining 87% of its initial capacity. In comparison, NMO electrode capacity drops rapidly during cycling, retaining only 24% after 200 cycles. These clearly demonstrate that low ionic potential medium‐entropy doping enhances the material's capacity and energy density while improving its cycling stability and rate performance. Note, the initial capacity increase in both samples can be attributed to the anionic redox reaction activation processes [39, 40]. Figure 2h and Table S5 compare the capacity and cycle life of recently reported cathode materials, highlighting the advantages of MEO. These results show that the low ionic potential medium‐entropy design significantly improves electrochemical performance, making MEO a promising cathode material for SIBs.

Hard carbon was used as an anode material to assemble full cells, with its electrochemical and cycling properties presented in Figure S13. The MEO cathode was coupled with the hard carbon anode by adjusting the N/P ratio to 1.02:1. The anode was presodiated in a Na‐metal half‐cell for one cycle. The first charge and discharge profiles of the full‐cell at 0.1 C (Figure S14) had a sloping curve‐type shape and high reversibility. The full‐cell achieves a reversible capacity of 194.3 mAh g^−1^ at 0.1 C (based on the positive electrode mass). The rate capability of the full‐cell is shown in Figure 2i. It indicates a satisfactory rate capability with a capacity retention of 45.2% as the current density increased from 0.1 to 5 C. Figure 2j demonstrates the excellent cycling stability of the full‐cell, maintaining a capacity retention of 85.9% even after 50 cycles at 1 C. The Ragone plot (Figure 2k), derived from the rate performance data, shows an energy density of 568.8 Wh kg^−1^ at 47.3 W kg^−1^ and a power density of 2245.5 W kg^−1^ at 247 Wh kg^−1^. In comparison with other recently reported sodium‐ion full cells, it exhibits significant superiority in both energy and power densities. These exceptional electrochemical results confirm the strong applicability of MEO cathodes.

### 2.3 Charge Compensation

**FIGURE 3:**
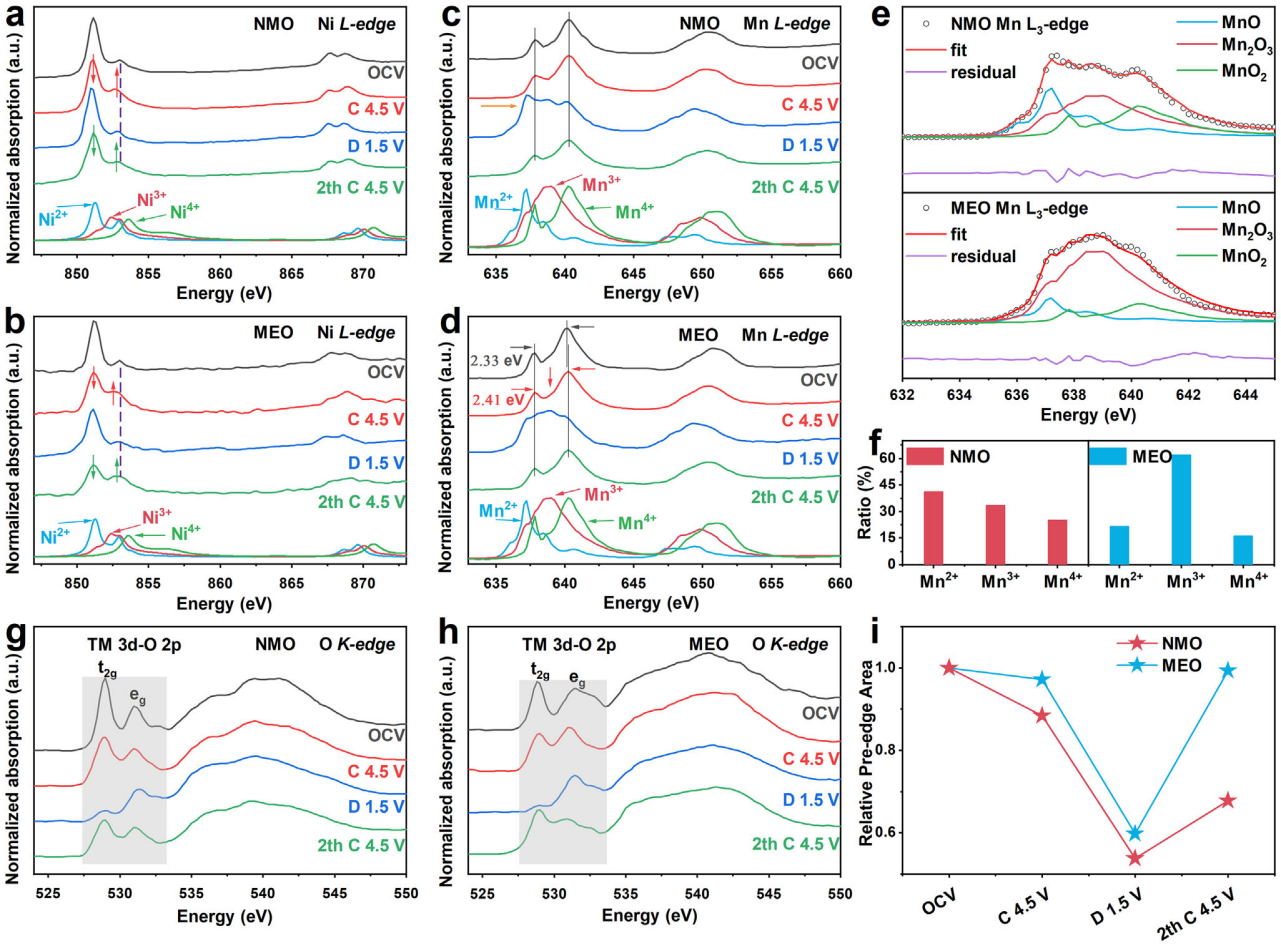
Electronic structure evolution. Ex situ Ni *L‐edge* spectra of (a) NMO and (b) MEO electrodes at different states. Bottom: Ni *L‐edge* XAS spectra of reference compounds—calculated spectra of Ni^2+^, Ni^3+^, and Ni^4+^ in an octahedral crystal field [51]. Ex situ Mn *L‐edge* spectra of (c) NMO and (d) MEO electrodes at different states. Bottom: Mn *L‐edge* XAS spectra of reference compounds—MnO (Mn^2+^), Mn_2_O_3_ (Mn^3+^), and MnO_2_ (Mn^4+^); orange arrows mark Mn disproportionation reaction. (e) Fitted spectra of the Mn *L_3_ edge* XAS spectra. (f) Evolution of fractional components of Mn^2+^, Mn^3+^, and Mn^4+^ obtained from spectral fitting at the 1.5 V discharge state for NMO and MEO electrodes. *Pre‐edge* of O *K‐edge* XAS spectra of (g) NMO and (h) MEO samples at different states. (i) The areas of the pre‐edge shaded regions for NMO and MEO.

The charge compensation mechanism of NMO and MEO was investigated by synchrotron X‐ray absorption spectroscopy (XAS) measurements for different charge and discharge states during the initial cycle. Figure 3a,b shows the corresponding Ni *L‐edge* spectra. Both samples exhibit similar spectra changes during the charging and discharging processes. The peak intensity at 851.3 eV decreases, while that at 853.0 eV increases and shifts to lower energy. During subsequent discharge, the spectrum reverts to its pristine state, indicating that Ni ions in both materials undergo the Ni^2+^/Ni^3+^ redox reaction [41, 42].

The Mn *L‐edge* spectrum of MEO (Figure S15) shows a 0.08 eV downward shift in the *e*_g_^*^ orbital and an emerging peak at 638.9 eV, both characteristic of Mn^3+^, which was further quantified by linear fitting. During the first charge process, the Mn *L‐edge* of NMO remains almost unchanged, whereas that of MEO shifts entirely to Mn^4+^ (Figure 3c,d). However, significant differences emerge during the discharge process. After discharge, part of Mn is reduced to the Mn^3+^ valence state. To determine the Mn valence percentages as a function of the charge state, the XAS spectral linear combination fitting method was employed for the samples discharged to 1.5 V (Figure 3e,f). NMO displays distinct spectral characteristics of Mn^2+^, which signals that a disproportionation reaction (2 Mn^3+^ → Mn^2+^ + Mn^4+^) caused by Jahn–Teller effect has occurred [43, 44]. The production of Mn^2+^ usually leads to structural degradation and manganese dissolution. These effects can impact the electrochemical activity of the cathode material, reduce its working voltage, and decrease its long‐term stability [45]. Post cycling ICP‐OES analysis (see Table S6 and Figure S16) shows that the leaching amounts of transition metals (including Mn, Ni, and others) from the NMO electrode are several times greater than those from the MEO electrode. The markedly lower presence of Mn^2+^ in MEO, both in the discharged electrode and in the electrolyte, underscores the role of the entropy‐stabilized structure in mitigating Mn dissolution. This stabilization helps to maintain the structural integrity of the material, thereby suppressing the voltage decay and the enhancing the cycling stability [46].

Figure 3g,h shows the O *K‐edge* spectra of NMO and MEO. The pre‐edge peaks before 535.0 eV are associated with the electronic transition from O 1s to O 2p–TM 3d hybridization states. Typically, the t_2g_ band corresponds to the lower energy peak near 529 eV, while the e_g_ band corresponds to the higher energy peak around 531.5 eV. The integral area under the pre‐edge peaks reflects the information about the holes and average effective charge on the O 2p–TM 3d hybridized orbitals [47, 48]. At the 4.5 V charged state, a distinct peak at 531 eV appears for both samples, widely recognized as the signature peak of oxidized oxygen [49]. The integrated intensity of this peak changes upon charge/discharge, reflecting the reversible formation and annihilation of O 2p hole states. Figure 3i displays the integrated areas of the pre‐edge peak (shaded region) for NMO and MEO. MEO exhibits more electron holes on oxygen atoms compared to NMO at both the 1st C4.5 V (0.97 vs. 0.88) and the 1st D1.5 V state (0.60 vs. 0.54). This striking difference confirms that the oxidation of lattice oxygen in MEO is significantly enhanced [50]. Furthermore, comparing the area integration values between the 1st C4.5 V and 2nd C4.5 V states for each material reveals a reduction of electron holes on O^2−^ in NMO. This implies that certain O atoms are irreversibly oxidized in NMO and do not fully participate in the subsequent anionic redox reaction, thus reducing the reversibility of the anionic redox reaction. In contrast, for MEO, the electron hole on O atoms does not decrease from the 1st C4.5 V (0.97) to the 2nd C4.5 V (0.99) state, demonstrating a significantly enhanced reversibility of the anionic redox reaction in MEO sample.

### 2.4 Structural Evolution

**FIGURE 4:**
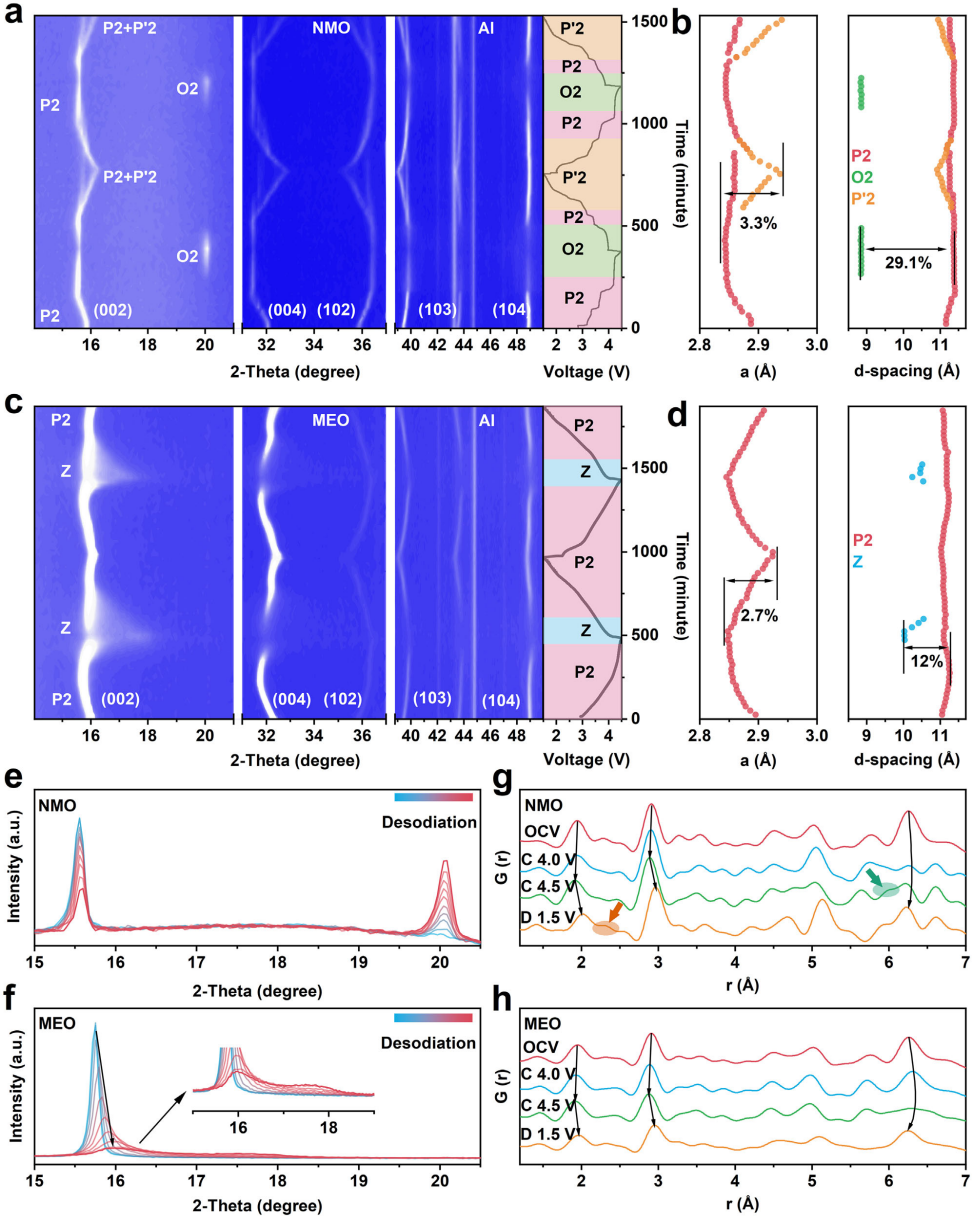
Crystal and local structure evolution. In situ XRD patterns of (a) NMO and (c) MEO during the first two cycle at 0.1 C. Evolution of the lattice parameters of (b) NMO and (d) MEO during the charge and discharge process. Enlarged in situ XRD patterns in the 2‐theta range of 15°–20.5° of (e) NMO and (f) MEO during the first charge at voltage region of 4.2–4.5 V. Ex situ PDF patterns of (g) NMO and (h) MEO at selected charge–discharge states.

In situ XRD analyses were conducted on both NMO and MEO samples for the initial 2 cycles. From the contour plots of the NMO (Figure 4a), the peaks belonging to the P2 phase continuously evolve upon charging and discharging. At ∼4.2 V, new diffraction features emerge at the Bragg positions corresponding to the O2 phase, accompanied by the attenuation of P2 reflections [52]. This transition is attributed to the increased interlayer repulsion and expansion during desodiation, which promote TM layer gliding and partially convert the P2 into O2 phase [4]. At voltages below ∼3.5 V, the O2 reflections vanish, indicating a reversible P2–O2 transition. At voltages below 3.0 V, however, strong Jahn–Teller distortion from high‐spin Mn^3+^ (*t*_2g_^3^‐*e*_g_^1^) induces severe lattice deformation of the TM octahedra, initiating a P2 to P'2 phase transition and the formation of a biphasic structure, which leads to significant anisotropic volume changes [53]. This transition is highly detrimental to structural integrity and cycle life in the Mn‐based cathodes. In the second cycle, similar phase transitions are observed, however, the onset voltages for the structural transitions during desodiation shift upward (to 3.7 and 3.4 V). It indicates that the structural change is not fully reversible, as the lattice distortion caused by the Jahn–Teller effect leaves residual strain, and raises the Na^+^ insertion energy barrier and thereby elevating the phase transition potentials in subsequent cycles [5, 54]. Quantitative analysis of the in situ XRD patterns (Figure 4b) shows that the a‐axis and interlayer distance exhibit a substantial contraction of approximately 3.3% and 29.1%, respectively, throughout the charging and discharging process. This inevitably leads to substantial and repetitive lattice strains, thereby inducing mechanical fractures that compromise the cyclic stability of the NMO electrode [55]. In contrast, MEO exhibits superior structural robustness. The in situ XRD contour plot (Figure 4c) shows no formation of new reflections, implying suppression of both P2–O2 and P2–P′2 transitions. Comparative peak stacking profiles (Figure 4e,f) further demonstrate that while NMO undergoes P2 peak fading and O2 peak emergence at high voltages, MEO only exhibits mild peak broadening without phase separation. This suggests a transition from P2 to a Z phase in MEO, which retains partially unslipped TM slabs, minimizing internal energy fluctuation and improving structural stability [11]. The distinct peak at 2θ ≈16° confirms that the O/P intergrowth in MEO predominantly maintains the P‐type stacking, with only limited layer sliding [56, 57]. The contraction along the a‐axis and interlayer distance in MEO is also significantly reduced (∼2.7% and ∼12%, respectively; Figure 4d), which mitigates internal strain accumulation and suppresses crack formation, contributing to enhanced cycling durability.

Ex situ PDF measurements further corroborate these results. As shown in Figure 4g,h. NMO shows pronounced shifts in intra‐layer atomic correlations during cycling, reflecting TM─O octahedral distortions driven by TM redox activity (Figure S17) [58]. Upon discharge to 1.5 V, a new peak at ∼2.3 Å emerges, characteristic of the P′2 phase [58], arising from Jahn–Teller‐induced distortion that splits the TM─O bond lengths into four short (∼2.0 Å) and two long (∼2.3 Å) bonds. In contrast, MEO shows negligible variation in intra‐layer peaks throughout cycling. At low voltage, Jahn–Teller distortions are effectively suppressed, in line with XAS observations. The nearest inter‐layer TM─TM distance in the P2 structure is ≈5.6 Å, overlapping with the intra‐layer TM─TM peak so it cannot be clearly distinguished, as shown in Figure S17. The first resolved inter‐layer correlation appears at ∼6.3 Å, corresponding to the next‐nearest TM─TM pairs across the inter‐layer gap. For NMO, a new peak at ∼6.0 Å emerges in the high‐voltage profiles, which is attributed to the inter‐layer TM─TM distance of an O2 layer, consistent with in situ XRD. By contrast, MEO shows no O2 signatures at high voltage, further confirming that the medium‐entropy design suppresses the structural phase transition.

The long‐term structural integrity of NMO and MEO was systematically studied. XRD patterns (Figure S18) show pronounced peak broadening and shifting for NMO after 100 and 200 cycles, signaling severe loss of crystallinity. In contrast, MEO maintains sharp diffraction peaks, demonstrating exceptional structural integrity [59]. This disparity is further corroborated by TEM (Figure S19). TEM images show severe microcracks in NMO particles after 200 cycles, whereas MEO particles remain intact. FFT patterns analysis confirm partial conversion of NMO into an electrochemically inactive rock‐salt phase (Fm‐3m), while MEO preserves the original layered structure without detectable rock‐salt domains [60].

This multi‐dimensional structural evolution underscores the critical role of medium‐entropy doping in stabilizing the P2‐type framework. In MEO, the multi‐element doping strategy constructs robust TMO_6_ units and a stable lattice oxygen framework within the TMO_2_ slab. This robust architecture effectively suppresses TM layer gliding, mitigates Jahn‐Teller distortion, and inhibits the cooperative failure of the NaO_2_ and TMO_2_ slabs. Consequently, the high‐voltage P2‐O2 phase transition and associated c‐axis contraction are markedly inhibited, enabling stable cycling at 4.5 V.

### 2.5 Discussions

**FIGURE 5:**
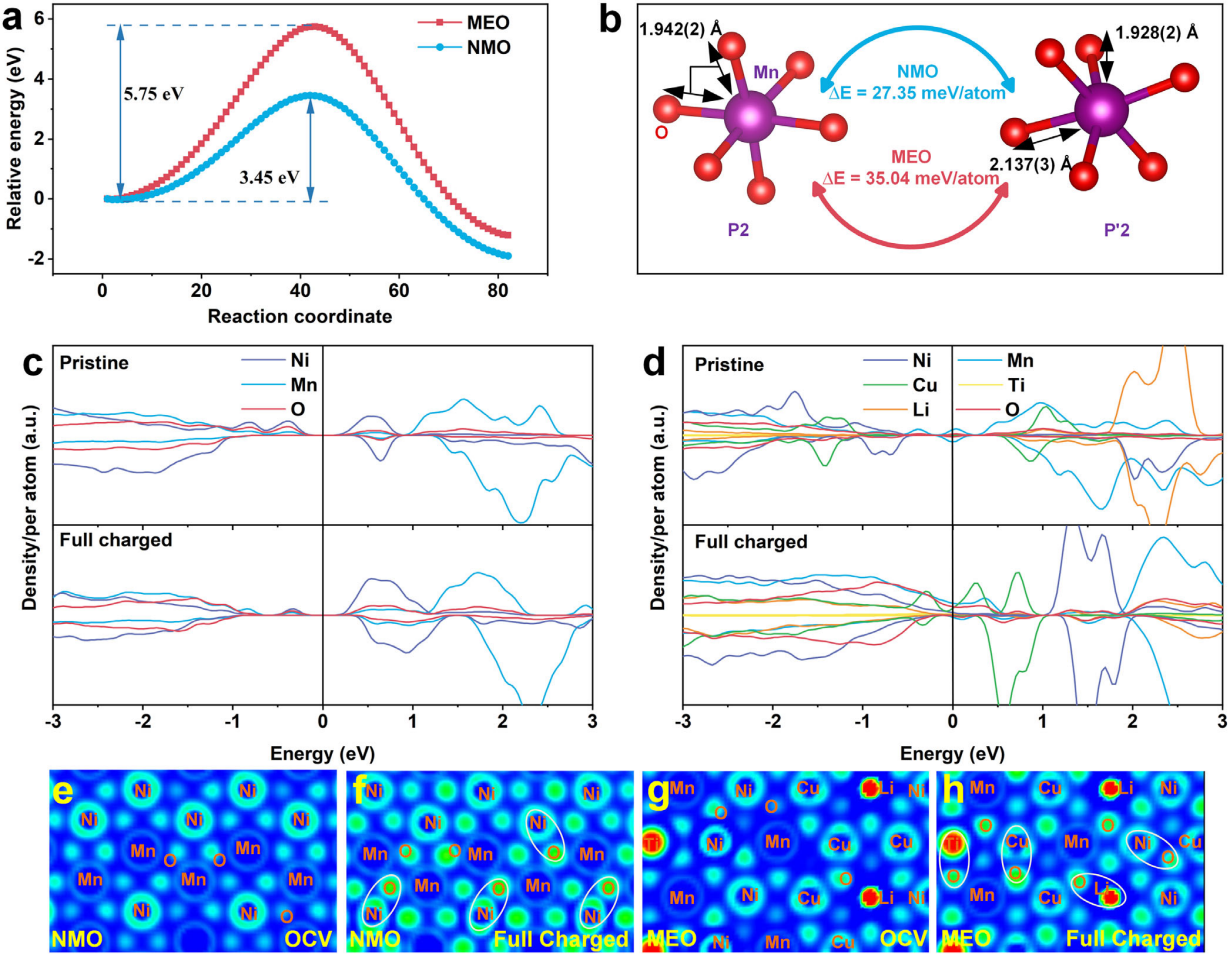
Density functional theory analysis. (a) Stacking fault energy of TM slab gliding profiles of NMO and MEO. (b) Phase transition energy of NMO and MEO form P2 to P′2. Projected density of states (PDOS) profiles of (c) NMO and (d) MEO at different states. Electron localization function images of (e) NMO and (g) MEO and the respective desodiation states (f) and (h).

The effect of medium‐entropy doping on TM slab gliding was further investigated by evaluating the stacking fault energies for MEO and NMO following the removal of Na^+^ ions final state using density functional theory (DFT) calculations. Figure S20 illustrates TM slab gliding configurations in the two samples. Each stacking fault energy profile exhibits two energy minima, corresponding to the total energy difference of the P2 and the O2 phase relative to the P2 phase (Figure 5a). The initial minimum represents the P2 phase of MEO/NMO, while the second (lower) minimum identifies the O phase as the thermodynamically favored state. The stacking fault energy barrier of MEO (5.75 eV) is 2.3 eV higher than NMO's (3.45 eV), indicating that MEO more effectively suppress the sliding of TM plates and thereby inhibit the transition from P‐type to O‐type stacking. Furthermore, the phase transition energies from P2 phase to P′2 phase after discharge are 35.04 and 27.35 meV/atom for MEO and NMO, respectively (Figure 5b; Figure S21). The higher barrier in MEO demonstrates that the medium‐entropy strategy markedly suppresses TM‐layer gliding and Jahn–Teller distortion, thereby exerting an obvious effect on stabilizing the structural changes during the Na deintercalation process.

To gain a deeper understanding of the effect of medium‐entropy doping on oxygen redox reactions, projected density of states (PDOS) and electron localization function (ELF) calculations were carried out for pristine and desodiated MEO, NMO. The corresponding crystal structures are illustrated in Figure S22. The PDOS for MEO and NMO are shown in Figure 5c,d. In pristine NMO, electronic states near the Fermi level (*E*_F_) are primarily attributed to Ni 3d, confirming Ni^2+^/Ni^3+^ as the dominant redox couple. For MEO, electronic states near *E*_F_ are dominated by Mn 3d owing to Mn^3+^, so Mn^3+^ oxidizes first, followed by Ni^2+^ and Cu^2+^, providing multiple redox centers. Upon desodiation, MEO accumulates more O 2p holes above *E*_F_ than NMO, demonstrating a greater contribution of lattice oxygen to charge compensation. Na─O─Li configuration introduces non‐bonding O 2p states that enhance oxygen redox activity, consistent with experiments. ELF calculations were used to analyze electron distributions within NMO and MEO (Figure 5e–h). In pristine NMO and MEO (Figure 5e,g), electrons were evenly distributed around TM─O bonds. Upon desodiation, in NMO, only the charge density distribution of O ions around Ni ions is significantly enhanced, resulting in a single and localized oxygen oxidation center (Figure 5f). From a thermodynamic perspective, due to the single and concentrated oxidation center of oxygen, oxygen ions are prone to excessive oxidation, promoting the formation of O_2_ [61]. In contrast, MEO exhibits multiple oxygen oxidation centers, featuring dispersed oxygen redox reactions. This prevents the instability of the oxygen lattice and avoids excessive oxygen oxidation (Figure 5h). These results prove that doping multiple low ionic potential elements improves the degeneracy of the O 2p states, thereby enhancing the activity and stability of high voltage oxygen redox reactions.

## 3 Conclusions

In conclusion, we systematically investigated a medium‐entropy P2‐type oxide, Na_0.8_Li_0.1_Ni_0.1_Cu_0.1_Ti_0.1_Mn_0.6_O_2_, co‐doped with multiple low ionic potential elements. Such doping enhances the degeneracy of O 2p states, thereby activating high‐voltage oxygen redox reactions while simultaneously suppressing unfavorable phase transitions. The enlarged TMO_2_ slab spacing stabilizes the layered framework and mitigates cation migration. As a result, the electrode delivers a high reversible capacity of 223.7 mAh g^−1^ and an energy density of 616.3 Wh kg^−1^, together with excellent rate capability (129.6 mAh g^−1^ at 5 C) and robust cycling stability (87% retention after 200 cycles). This medium‐entropy design provides a synergistic optimization of composition and structure, significantly enhancing capacity, ion transport kinetics, and structural durability. The demonstrated strategy effectively overcomes long‐standing challenges of Mn‐based layered cathodes, offering a practical route toward next‐generation high‐capacity, high‐stability sodium‐ion batteries.

## Conflicts of Interest

The authors declare no conflicts of interest.

## Supporting information




**Supporting File**: advs73511‐sup‐0001‐SuppMat.docx.

## Data Availability

The data that support the findings of this study are available from the corresponding author upon reasonable request.
