# Supplementary material for: Enhanced Oxygen Redox Activity and Structure Stability of P2‐Type Manganese‐Based Cathodes Through Medium‐Entropy Strategy
Source: Adv Sci (Weinh). 2026 Jan 4;13(13):e18795. doi: 10.1002/advs.202518795 (PMC12955933; doi:10.1002/advs.202518795)
Supplement: Supplementary file 1 — Supporting File: advs73511‐sup‐0001‐SuppMat.docx. [file ADVS-13-e18795-s001.docx]

Supporting Information

**Enhanced Oxygen Redox Activity and Structure Stability in P2-Type Manganese-Based Cathodes through Medium-Entropy Strategy**

*Dongxiao Wang, Yuxuan Liu, Zihao Wang, Wei Su, Xingguo Qi, Huican Mao*, Kan Zhang, Shigang Lu, Bingkun Guo, and Yingchun Lyu**

D. Wang, Y. Liu, Z. Wang, W. Su, Prof. B. Guo, Prof. Y. Lyu

Materials Genome Institute, Shanghai University, Shanghai 200444, China

E-mail: yclyu@shu.edu.cn

Dr. X. Qi

HiNa Battery Technology Co., Ltd, Liyang 213300, China

Prof. H. Mao, Prof. K. Zhang

Department of Materials Science and Key Laboratory of Automobile Materials, MOE, Jilin University, Changchun 130012, China

E-mail: hcmao@jlu.edu.cn

Prof. S. Lu

College of Sciences and Institute for Sustainable Energy, Shanghai University, Shanghai 200444, China

**Experimental Section**

*Material Preparation*

P2-Na_0.67_Ni_0.33_Mn_0.67_O_2_ and P2-Na_0.8_Li_0.1_Ni_0.1_Cu_0.1_Ti_0.1_Mn_0.5_O_2_ were prepared by a solid-state reaction. The starting precursors of sodium carbonate (98%, Alfa Aesar), lithium carbonate (98%, Alfa Aesar), nickel oxide (99.9%, Alfa Aesar), cupric oxide (99%, Alfa Aesar), titanium oxide (99%, Alfa Aesar) and manganese sesquioxide (99%, Alfa Aesar) were mixed by stoichiometric ratio. The starting materials were ball-milled with ethanol at 500 rpm for 8 h by using a planetary ball mill. The mixture was dried and pelletized, then heated to 900 °C in air at a temperature increase rate of 2 °C min^−1^, and the temperature was held for 15 h. Cool to room temperature To determine the optimal sintering temperature, MEO powders were also calcined at 600, 650, 700, 750, 800, 850, 900, 950, 1000, 1050 and 1100 °C for 15 h under otherwise identical conditions. All calcined pellets were immediately transferred into a glovebox filled with argon and keep inside to avoid exposure to moisture in the air.

*Materials Characterization*

The specific ratio of Na, Li, Ni, Cu, Ti and Mn in the samples was measured by inductively coupled plasma atomic emission spectrometry (ICP-OES-Agilent 730). The NMO and MEO coin cells were disassembled in a glove box. The cycled NMO and MEO cathodes were washed three times with dimethyl carbonate (DMC) to remove residual electrolyte before further analysis. X-ray diffraction (XRD) patterns were measured using a PANalytical Empyrean diffractometer equipped with a Cu Kα radiation source (𝜆_1_ = 1.54060 Å, 𝜆_2_ = 1.54439 Å). The powder patterns were refined using GSAS Ⅱ software. The X-ray total scattering experiments were obtained on a PANalytical Empyrean outfitted with Ag Kα radiation (𝜆_1_ = 0.55941 Å, 𝜆_2_ = 0.56381 Å). The samples were loaded into a glass capillary (0.7 mm in diameter) and then sealed with ultraviolet glue. The total scattering data was collected from 2 to 140° for 10 h. The scattering signal of an empty glass capillary was measured as the background scattering. The obtained X-ray total scattering was transformed into the pair distribution function (PDF) data with the X’Pert HighScore Plus software.^[1, 2]^ The Q_min_ and Q_max_ used in the Fourier transformation were 0.4 and 20 Å^−1^, respectively. The sample morphology and composition characterizations were carried out with a scanning electron microscope (SEM, Hitachi SU8230) with an energy dispersive X-ray spectrometer (EDS). The high-resolution transmission electron microscopy measurements were performed with a transmission electron microscopy (TEM, JEM-2100F, JEOL) and high-angle annular dark field scanning transmission electron microscope (HAADF-STEM, JEOL ARM200F). The cycled electrodes were first sealed with polyimide tape in an Ar-filled glove box to avoid contact with air. O *K-edge* X-ray absorption spectroscopy (XAS) was performed at the BL02B02 beamline of the Shanghai Synchrotron Radiation Facility (SSRF). Mn and Ni *L-edge* XAS measurements were carried out at SSRF's BL08U1A beamline, and Mn *K-edge*, Ni *K-edge* XAS measurements at its BL16U1 beamline. The separator of the disassembled battery was dissolved to serve as the ICP testing sample for TM leaching after cycling.

*Electrochemical Measurements*

The electrochemical properties of the samples were investigated with CR2032 coin cells. The working electrodes were prepared by casting a slurry of active material, acetylene black, and polyvinylidene fluoride (PVDF) binder in a 7:2:1 weight ratio with N-methyl-2-pyrrolidone (NMP) as solvent coated onto an aluminum current collector. The coin cells were assembled in an argon-filled glove box with the cathode, dried at 100 °C under vacuum for 10 h, a pure sodium anode, a Whatman glass fiber separator, and electrolyte of 1 M NaClO_4_ dissolved in PC: EC: FEC (47.5:47.5:5). The active materials loading of electrode is 1.8 – 2.5 mg cm^−2^. These cells will be aged 2 h prior to electrochemical measurements. Galvanostatic charge-discharge tests were carried out on a LANHE CT2001A battery test system from 1.5-4.5 V at a rate of 0.1 C. The rate performance of the electrode was measured on a MACCOR 4200 battery test system.

*Computational Methods*

All the calculations are performed in the framework of the density functional theory with the projector augmented plane-wave method, as implemented in the Vienna ab initio simulation package.^[3]^ The generalized gradient approximation proposed by Perdew, Burke, and Ernzerhof is selected for the exchange-correlation potential.^[4]^ The long-range van der Waals interaction is described by the DFT-D3 approach.^[5]^ The cut-off energy for plane wave is set to 500 eV. The energy criterion is set to 10^−6^ eV in iterative solution of the Kohn-Sham equation. The K-mesh resolved in real space is 0.04 2π Å^-1^. All the structures are relaxed until the residual forces on the atoms have declined to less than 0.03 eV Å^-1^. The DFT+U method was implied to treat the strong on-site Coulomb interaction of localized electrons, which is not correctly described by LDA or GGA. The effective U-J values for Mn-3d, Ti-3d, Ni-3d, and Cu-3d were set to 2.4, 5.14, 5, and 6 eV, respectively, according to the previous works.^[6, 7]^


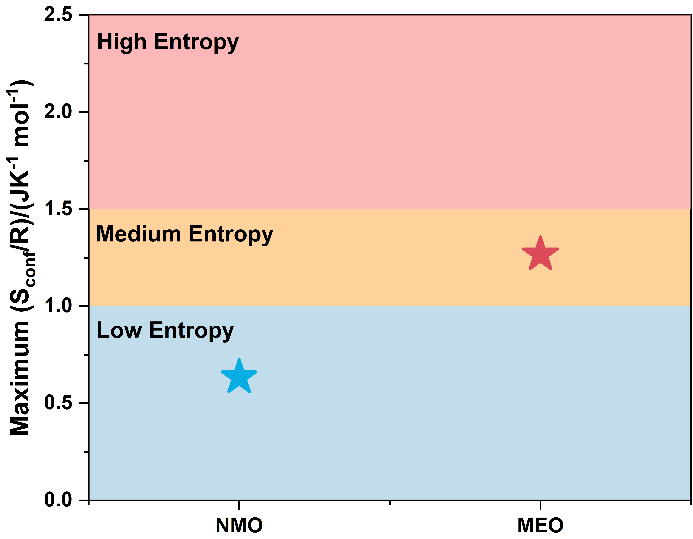


Figure S1. Maximum configurational entropy calculation for NMO and MEO.

For given materials, the conformational entropy per mol can be evolved by equation (1):

$\triangle S_{c\mathrm{onfig}}=-R\left[ \left( \sum_{i=1}^{N} x_{i}lnx_{i} \right)_{cation-site}+\left( \sum_{j=1}^{M} x_{j}lnx_{j} \right)_{anion-site} \right]$  (1)

where *R* is the gas constant, $x_{i}$ and$x_{j}$ are the mole fraction of elements occupying cation and anion site, N and M are the number of elements occupying the cation and anion site.^[8]^


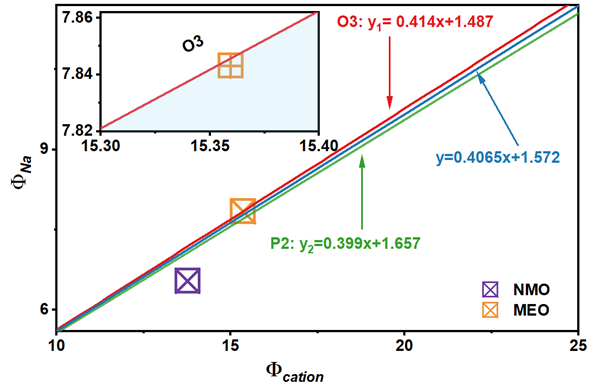


Figure S2. The cationic potential of the designed layered oxides, and corresponding illustration of P2-type and O3-type structures.^[1]^


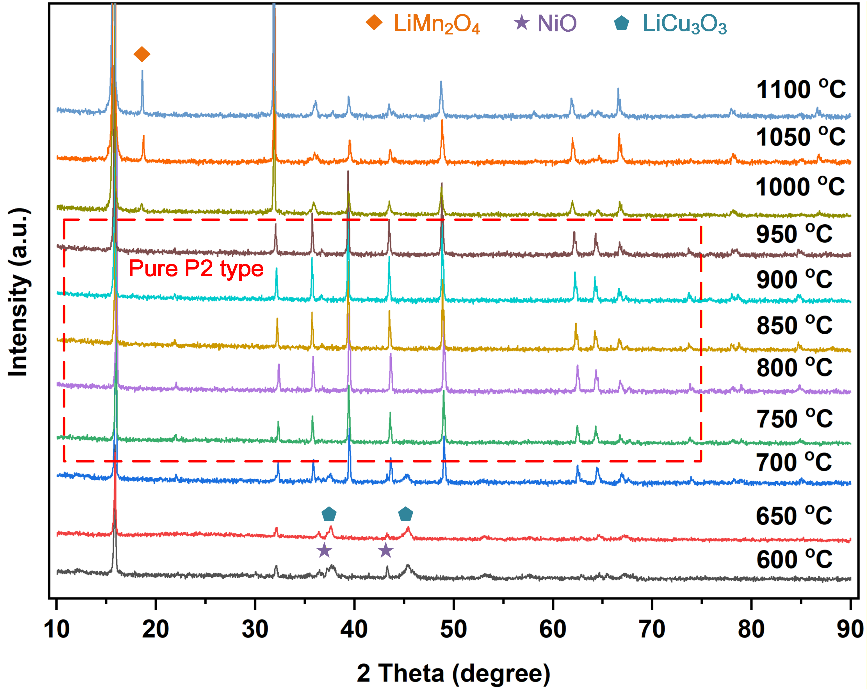


Figure S3. XRD patterns of MEO samples obtained at 600-1100 ℃ in air.


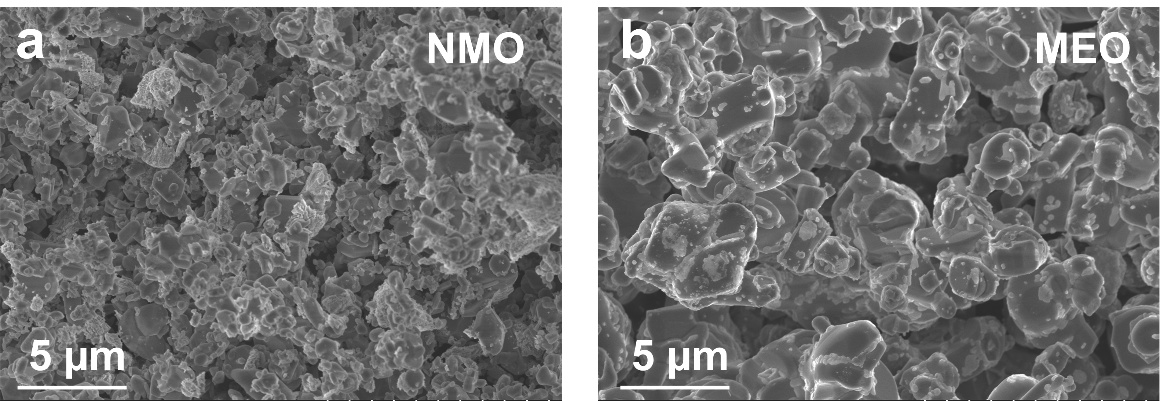


Figure S4. Scanning electron microscopy (SEM) images of a) NMO and b) MEO.


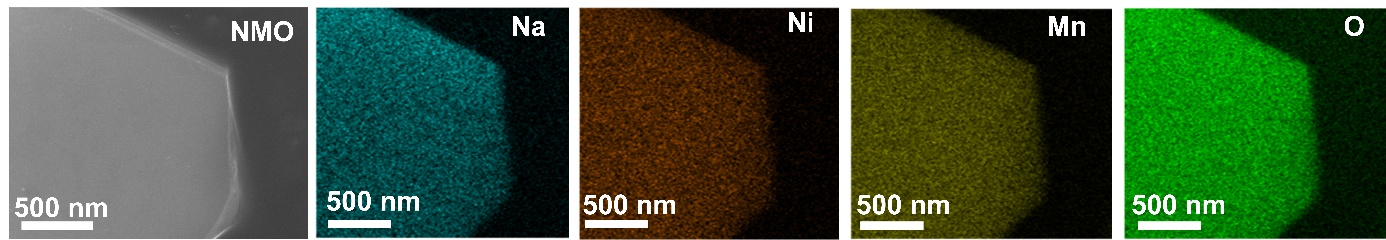


Figure S5. The SEM-energy dispersive spectroscopy (EDS) mappings of NMO.


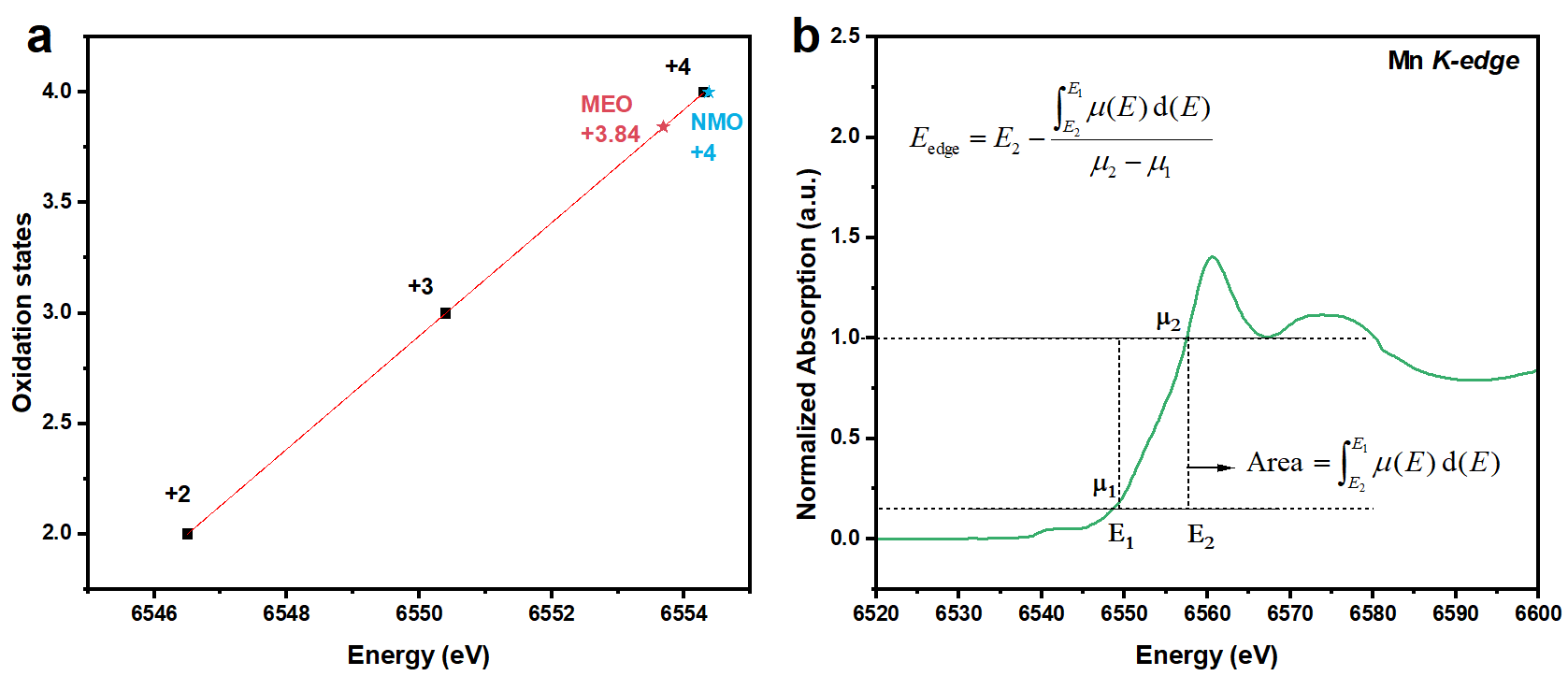


Figure S6. a) Linear relationship between the oxidation states of Mn and the *K-edge* positions determined by the integral method. The approximate Mn oxidation states of MEO and NMO can be deduced from the linear relationship between the oxidation states of reference and their edge positions obtained by the integral method. b) Detail of the XANES fitting. The average Mn oxidation states of the samples can be approximately deduced from this fitted line. Considering the effect of the variation in the shape of the absorption edge on the average edge energy due to the coordination environment change, the integral method is adopted and integration is performed between μ_1_ = 0.15 and μ_2_ = 1.0 on each normalized spectrum.


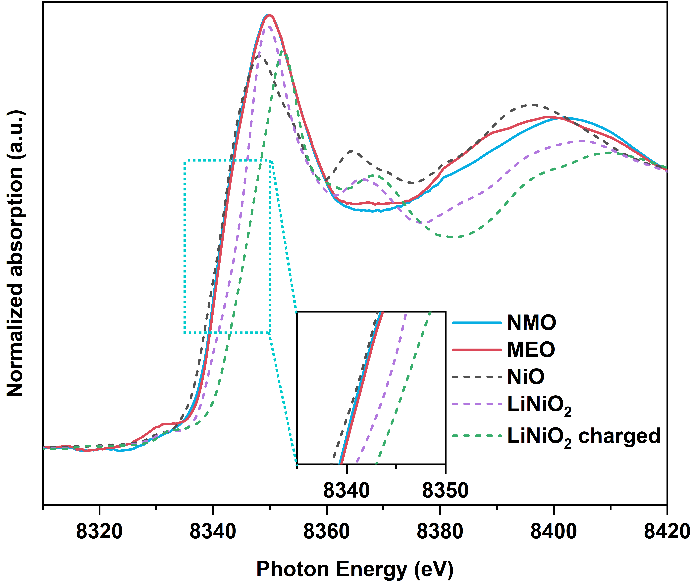


Figure S7. The Ni *K-edge* XANES spectra for the NMO and MEO samples with reference spectra of NiO, LiNiO_2_ and LiNiO_2_ charged sample.


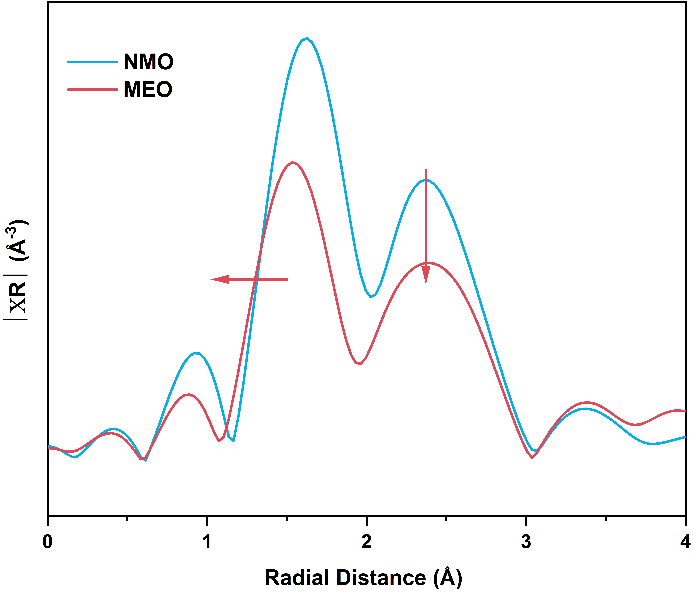


Figure S8. Ni *K-edge* FT-EXAFS spectra of NMO and MEO samples.


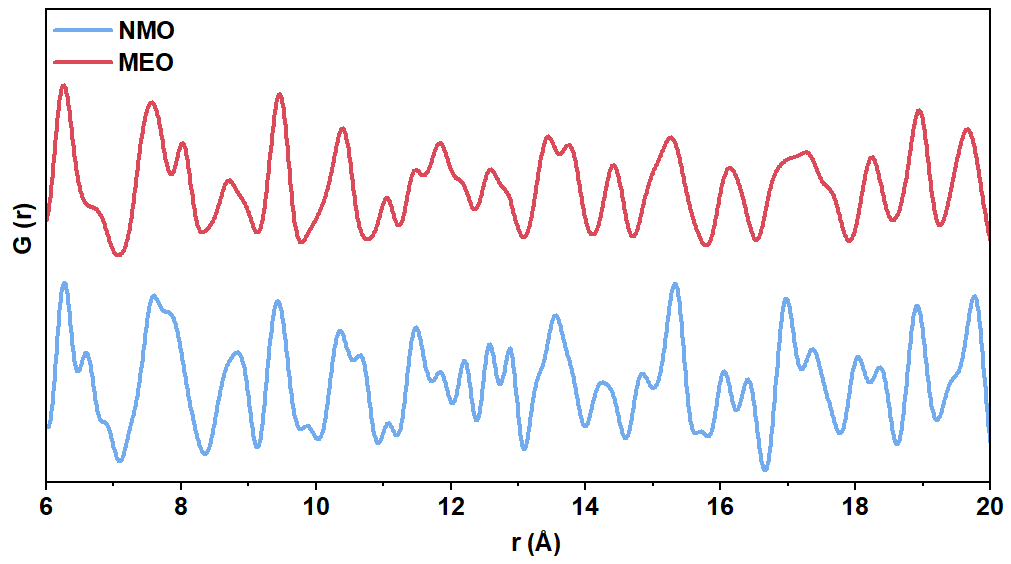


Figure S9. X-ray pair distribution function (PDF) of MEO and NMO in the long range (6-20 Å).


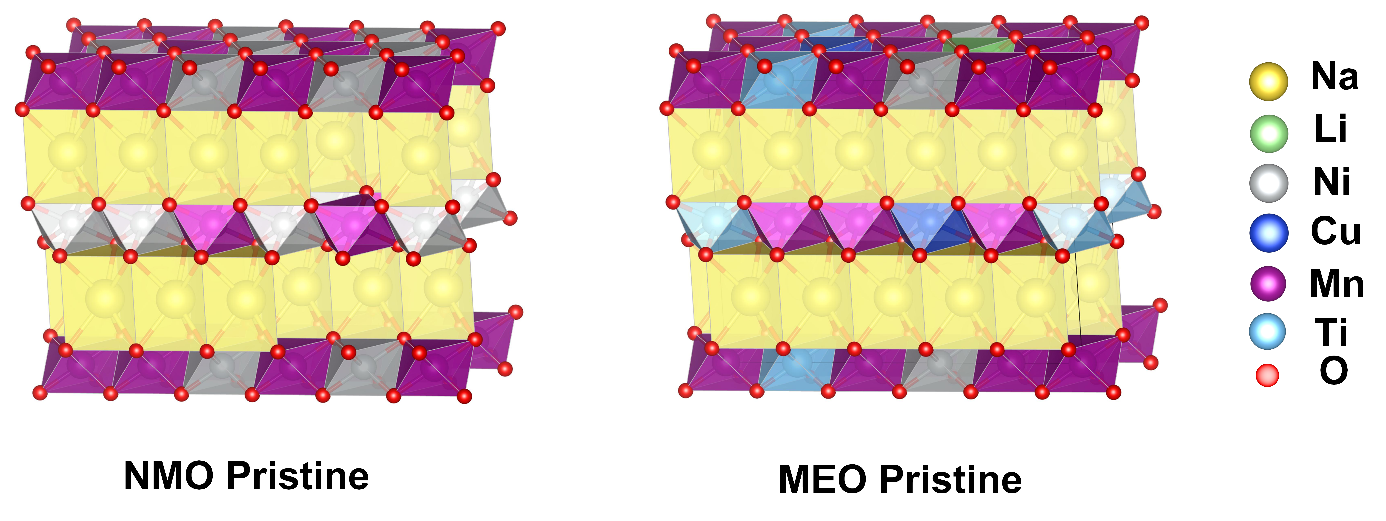


Figure S10. The DFT-relaxed structures of the NMO and MEO samples.


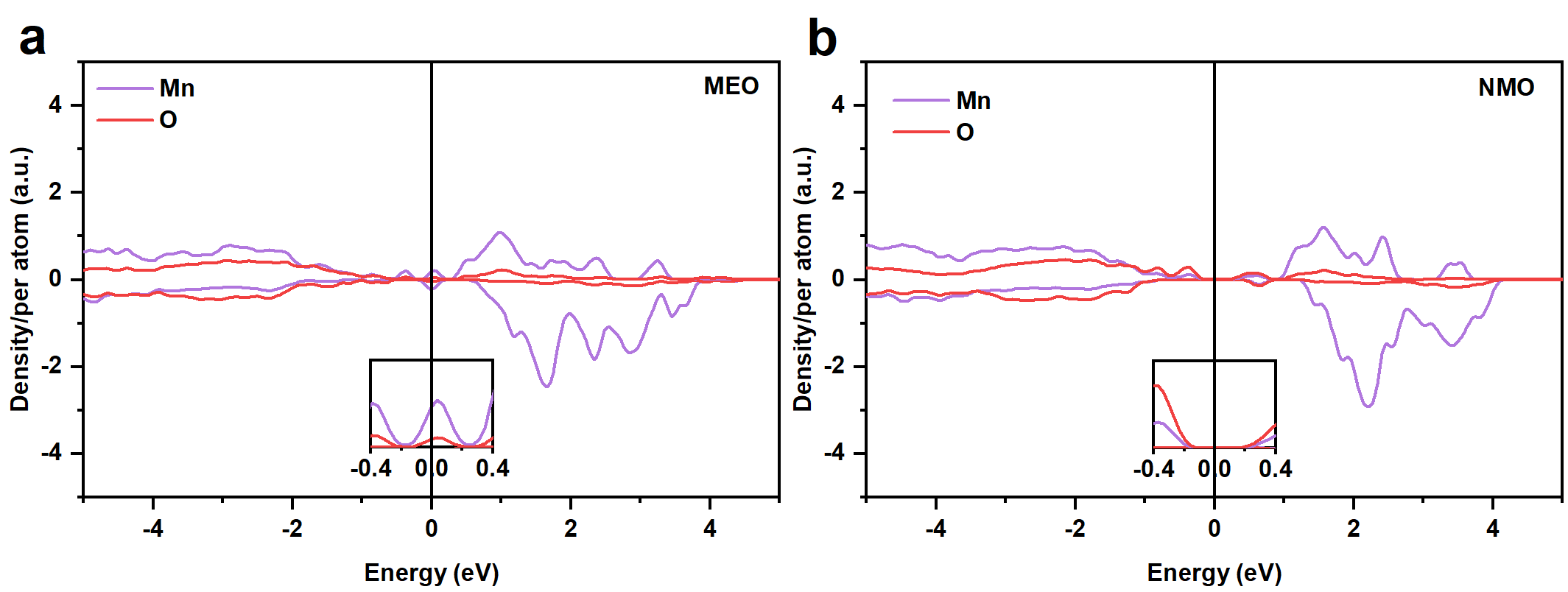


Figure S11. Projected density of states (PDOS) profiles of a) NMO and b) MEO samples.


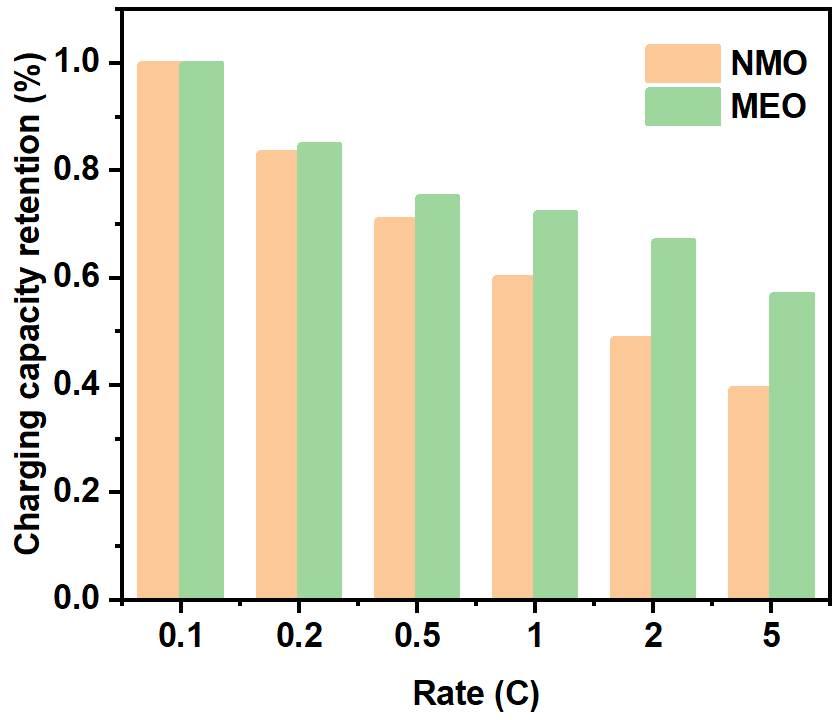


Figure S12. Comparison of charge specific capacity between NMO and MEO at different rate.


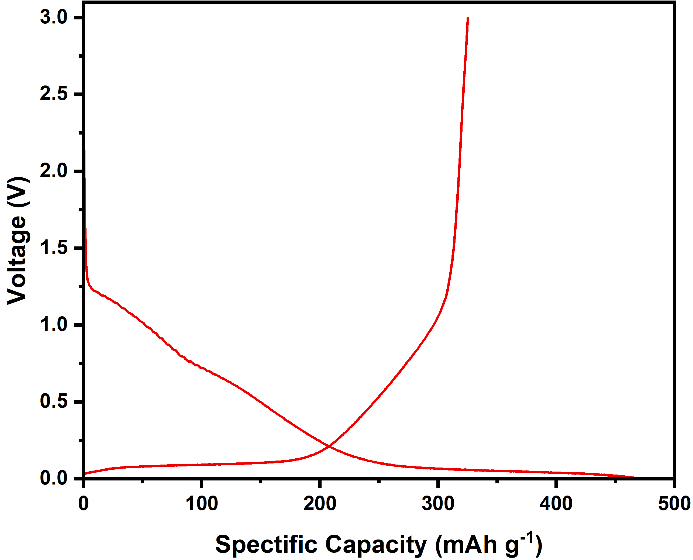


Figure S13. The first charge/discharge profiles of the hard carbon anode.


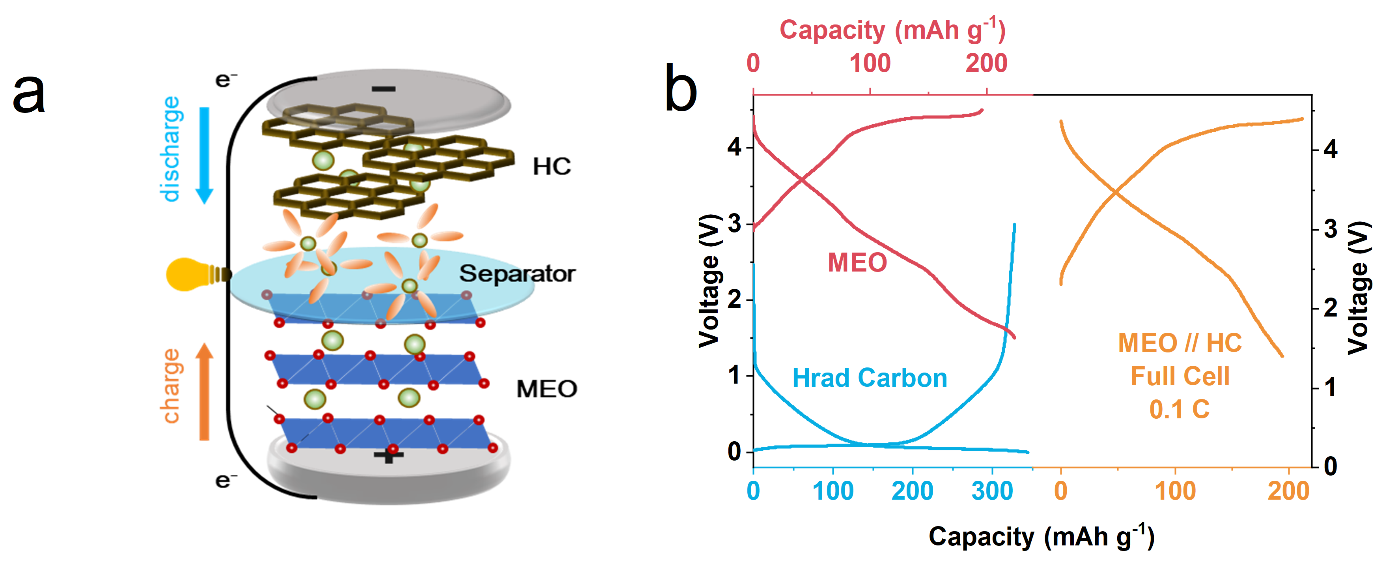


Figure S14. a) Schematic illustration of the MEO//hard carbon Na-ion full battery. b) Galvanostatic charge/discharge curves for MEO cathode (red), HC anode (blue), and MEO//HC full cell (yellow).


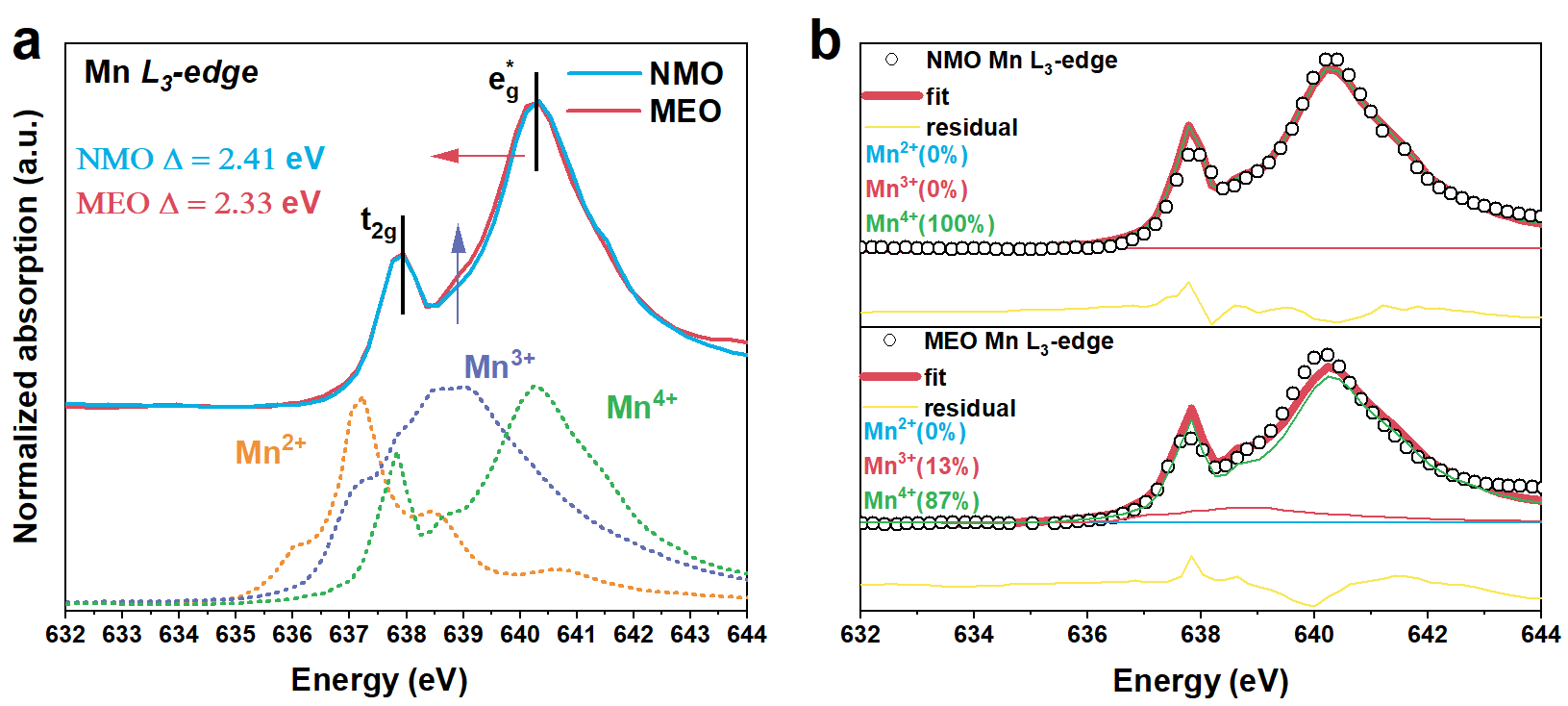


Figure S15. a) The Mn *L_3_-edge* XANES spectra for the as-prepared NMO and MEO samples and the manganese oxides. Bottom: Mn *L-edge* XAS spectra of reference compounds—MnO (Mn^2+^), Mn_2_O_3_ (Mn^3+^), and MnO_2_ (Mn^4+^). b) Fitted as-prepared NMO and MEO samples of the Mn *L_3_ edge* XAS spectra.


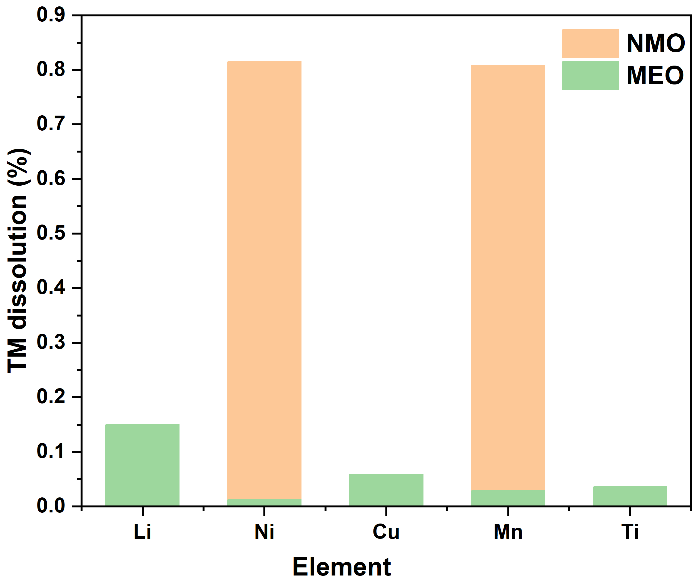


Figure S16. Li, Ni, Cu, Mn, and Ti dissolution ratio of NMO and MEO (after 100 cycles, 1.5-4.5V).


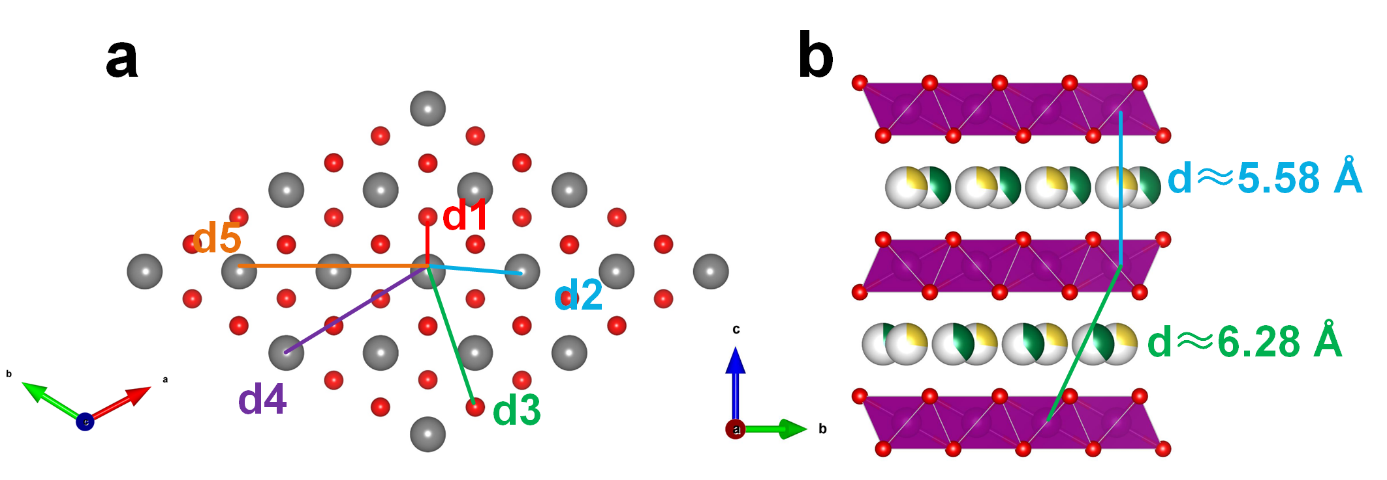


Figure S17. a) The in-plane atom pairs viewed along the c direction. The labels d1–d5 correspond to the first five peaks in the PDF G(r) patterns. Peaks d1–d5 at 1.93, 2.90, 4.50, 5.00, and 5.76 Å are assigned to the nearest TM–O bond, the nearest TM–TM bond, a sub-neighboring TM–O bond, a sub-neighboring TM–TM bond, and the third-nearest-neighbor TM–TM distance, respectively. b) The interlayer atomic distances viewed along a/b direction. The green and red arrows indicate the nearest and next nearest TM interactions between layers.


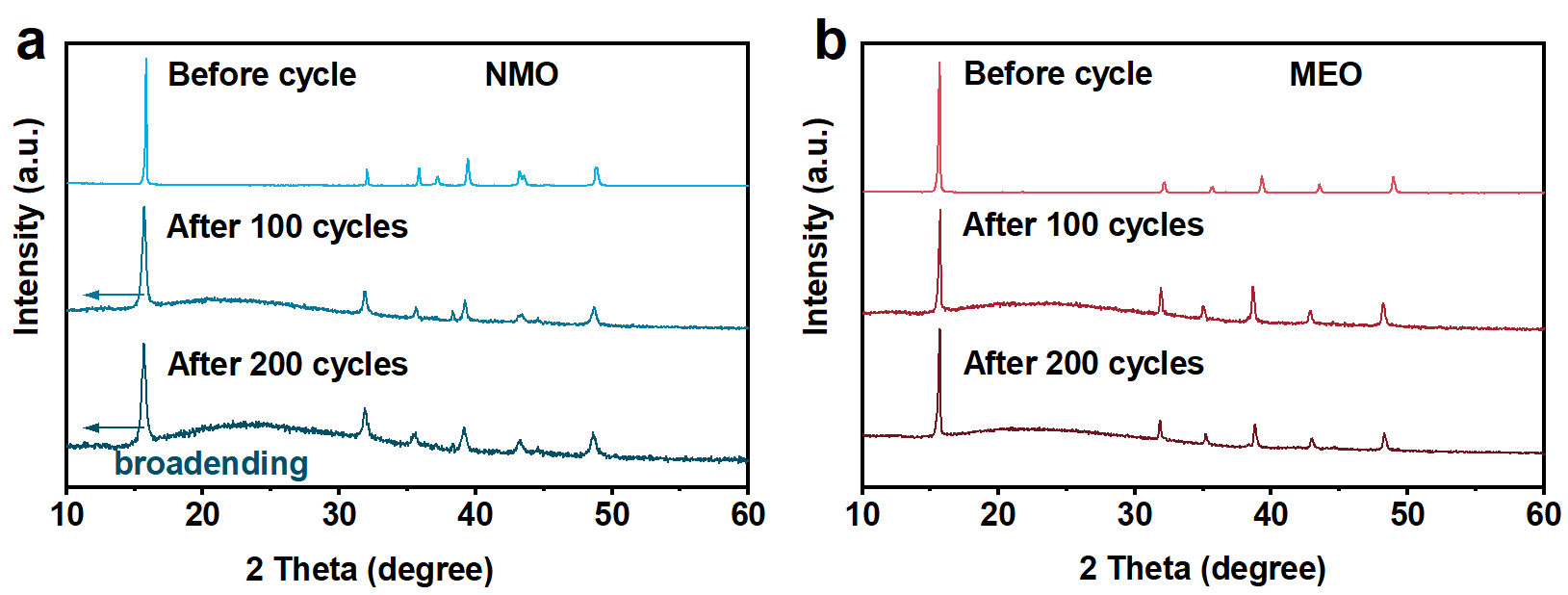


Figure S18. Ex situ XRD patterns of (a) NMO and (b) MEO electrodes after 100 and 200 cycles at 1 C. The broad bump located at 2θ ≈ 20–30° originates from the polyimide film used to protect the electrode from air exposure during measurement.


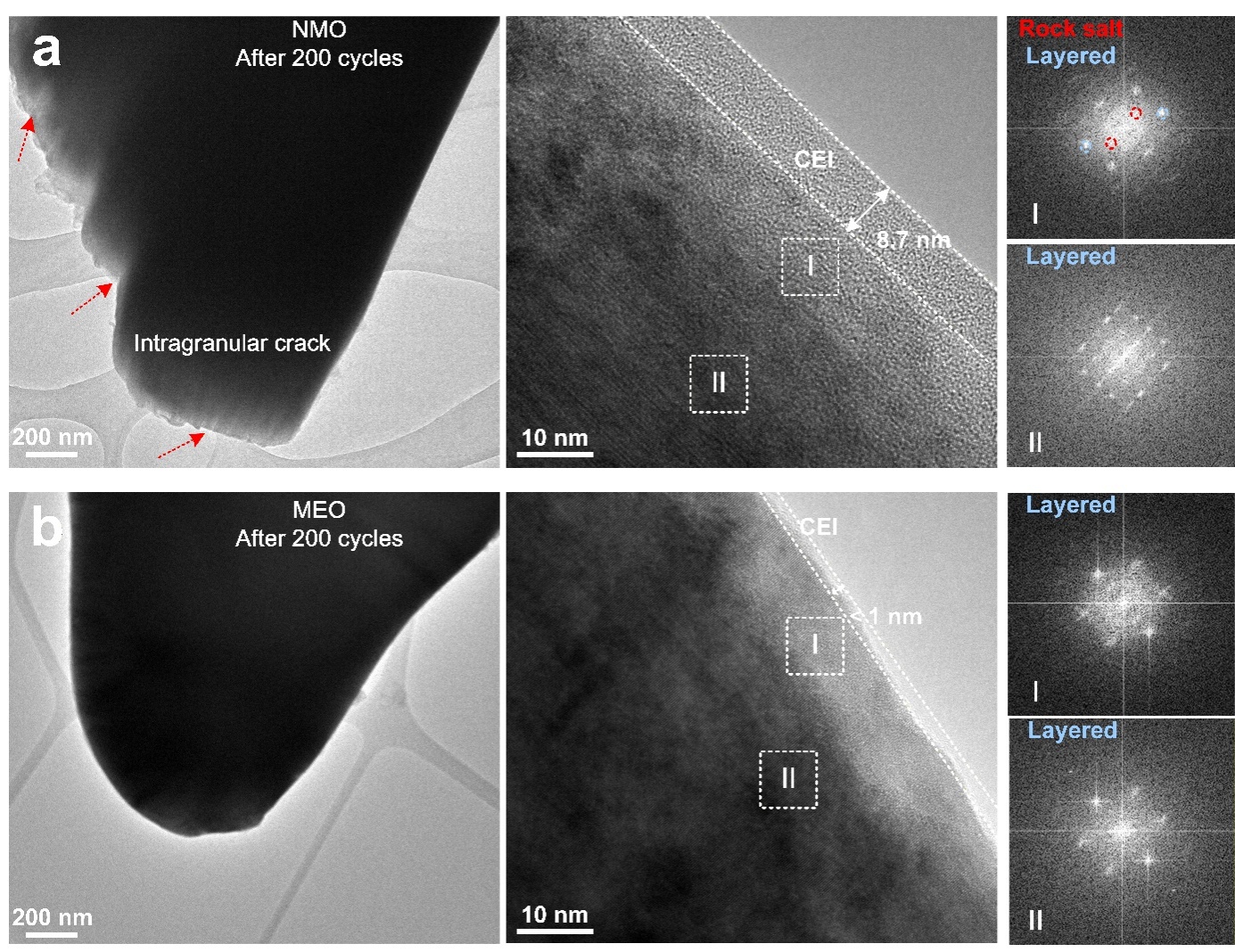


Figure S19. TEM images of cycled a) NMO and b) MEO after 200 cycles at 1 C.


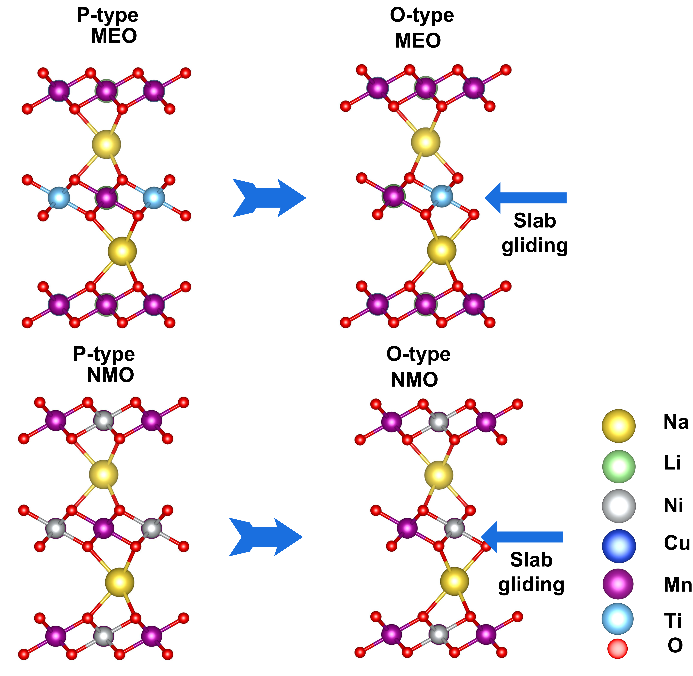


Figure S20. Schematic of transition metal (TM) slab gliding in a) NMO and b) MEO.


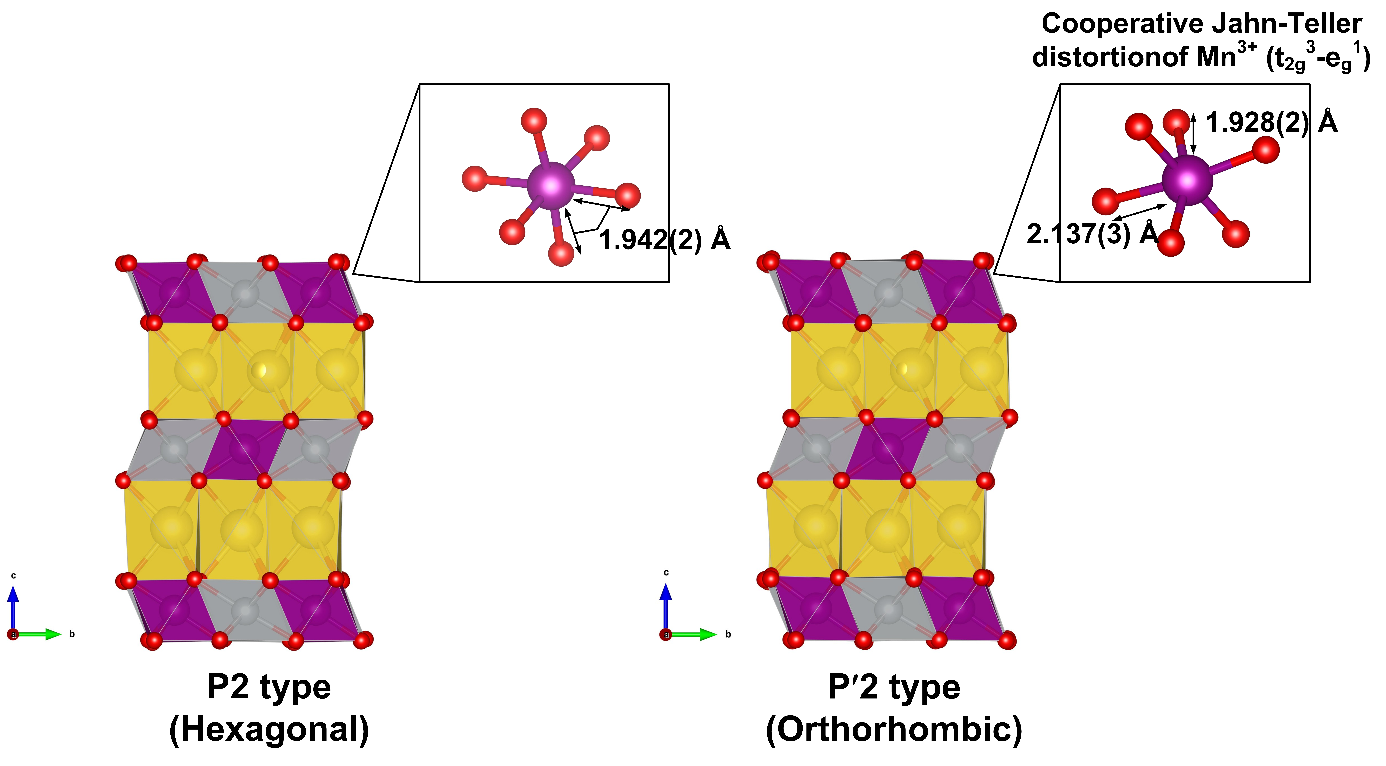


Figure S21. Schematic illustrations of P′2-type NMO and P2-type NMO, adopting an orthorhombic and a hexagonal unit cell, respectively.


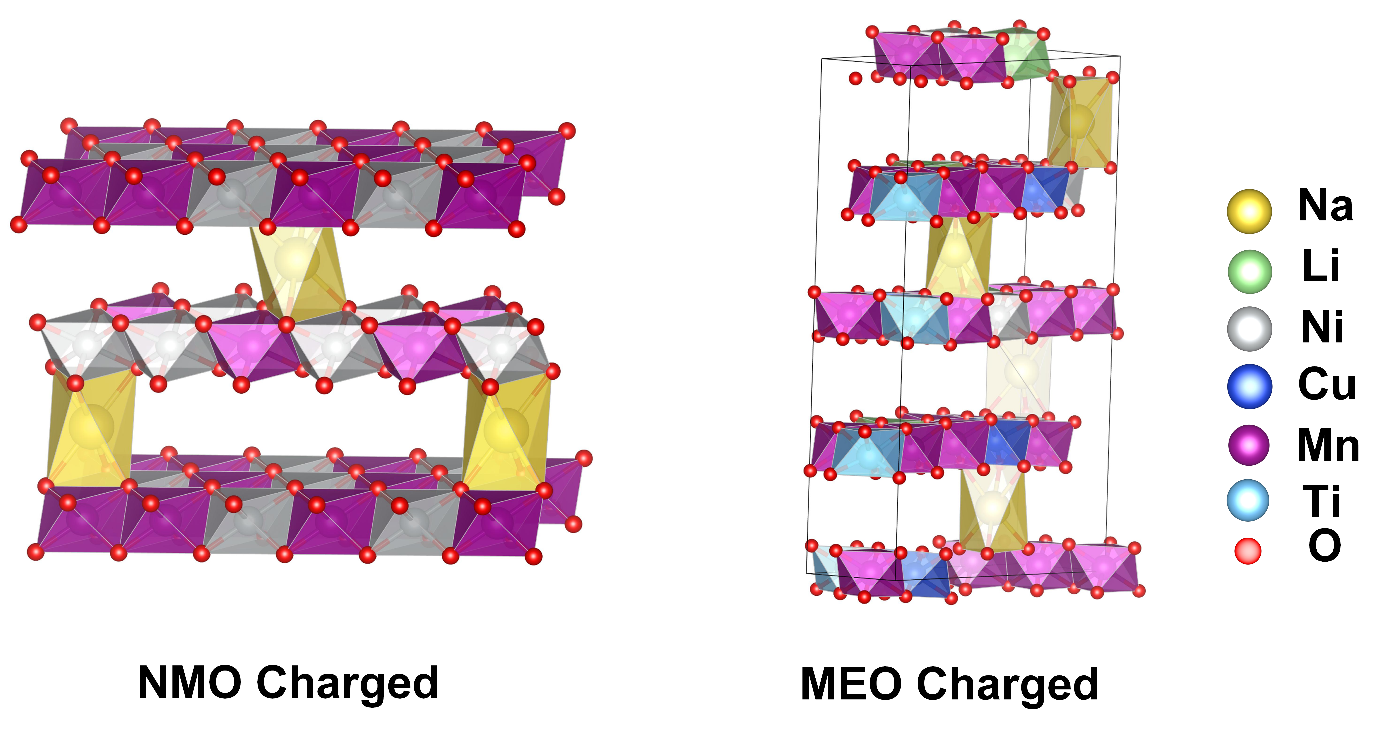


Figure S22. The DFT-relaxed structures of NMO and MEO desodiation states.

Table S1. Charge, ionic radius (nm) and ionic potential of the elements used in Na-ion layered oxides on the base of the oxidation states and atomic mass number.^[9]^

| Ion | Charge | Ionic radius (nm) | | Ionic potential (nm^-1^) | |
| --- | --- | --- | --- | --- | --- |
|  |  | High spin | Low spin | High spin | Low spin |
| Li | 1 | 0.0760 |  | 13.1579 |  |
| Mg | 2 | 0.0720 |  | 27.7778 |  |
| Fe | 2 | 0.0645 | 0.0550 | 31.0078 | 36.3636 |
| Ni | 2 | 0.0690 |  | 28.9855 |  |
| Cu | 2 | 0.0730 |  | 27.3973 |  |
| Al | 3 | 0.0535 |  | 56.0748 |  |
| V | 3 | 0.0640 |  | 46.8750 |  |
| Cr | 3 | 0.0615 |  | 48.7805 |  |
| Mn | 3 | 0.0645 | 0.0580 | 46.5116 | 51.7241 |
| Fe | 3 | 0.0645 | 0.0550 | 46.5116 | 54.5455 |
| Co | 3 | 0.0610 | 0.0545 | 49.1803 | 55.0459 |
| Ni | 3 | 0.0600 | 0.0560 | 50.0000 | 53.5714 |
| Ti | 4 | 0.0605 |  | 75.4717 |  |
| V | 4 | 0.0580 |  | 68.9655 |  |
| Cr | 4 | 0.0550 |  | 72.7273 |  |
| Mn | 4 | 0.0530 |  | 75.4717 |  |

To provide a descriptor for layered structure, cationic potential is defined to express the extent of the cation electron density and its polarizability, normalized to the anion ionic potential (2):

$\Phi=\bar{\Phi_{TM}}$ (2)

Where $\bar{\Phi_{\mathrm{TM}}}$ represents the weighted average ionic potential of TMs, defined as $\bar{\Phi_{TM}}=\sum\frac{w_{i}n_{i}}{R_{i}}$, $w_{i}$ is the content of ${TM}_{i}$ having oxidation state $n_{i}$ and radius $R_{i}$.

Table S2. ICP-AES results for NMO and MEO sample.

|  | Na | Li | Ni | Cu | Mn | Ti |
| --- | --- | --- | --- | --- | --- | --- |
| Na_0.8_Li_0.1_Ni_0.1_Cu_0.1_Mn_0.6_Ti_0.1_O_2_ | 0.796 | 0.098 | 0.102 | 0.098 | 0.597 | 0.105 |
| Na_0.67_Ni_0.33_Mn_0.67_O_2_ | 0.662 |  | 0.327 |  | 0.673 |  |

Table S3. Refined crystallographic parameters by Rietveld analysis for Na_0.8_Li_0.1_Ni_0.1_Cu_0.1_Mn_0.6_Ti_0.1_O_2._

Space groups: P6_3_/mmc, a= b= 2.8983(3) Å, c= 11.0865(8) Å, V= 80.65(4) Å^3^,

R_p_= 3.66%, R_wp_= 1.91%

|  | site | x | y | z | Occ. |
| --- | --- | --- | --- | --- | --- |
| Na1 | 2d | 0.6667 | 0.3333 | 0.25 | 0.17(9) |
| Na2 | 2b | 0.0000 | 0.0000 | 0.25 | 0.49(1) |
| Li1 | 2a | 0.0000 | 0.0000 | 0.0000 | 0.100 |
| Ni1 |  |  |  |  | 0.100 |
| Cu1 |  |  |  |  | 0.100 |
| Mn1 |  |  |  |  | 0.600 |
| Ti1 |  |  |  |  | 0.100 |
| O1 | 4f | 0.6667 | 0.3333 | 0.076(0) | 1.000 |

Table S4. Refined crystallographic parameters by Rietveld analysis for Na_0.67_Ni_0.33_Mn_0.67_O_2._

Space groups: P6_3_/mmc, a= b= 2.8880(0) Å, c= 11.1510(1) Å, V= 80.54(5) Å^3^,

R_p_= 5.99%, R_wp_= 2.71%

|  | site | x | y | z | Occ. |
| --- | --- | --- | --- | --- | --- |
| Na1 | 2d | 0.6667 | 0.3333 | 0.25 | 0.22(3) |
| Na2 | 2b | 0.0000 | 0.0000 | 0.25 | 0.44(7) |
| Ni1 | 2a | 0.0000 | 0.0000 | 0.000 | 0.333 |
| Mn1 |  |  |  |  | 0.667 |
| O1 | 4f | 0.6667 | 0.3333 | 0.078(1) | 1.000 |

Table S5. Performances of recently reported oxide-type cathode materials with fully occupied TM layers.

| Cathode material | Specific  capacity (mAh g^−1^) | Cycle Number (n) | Capacity retention (%) | Refs. |
| --- | --- | --- | --- | --- |
| Na_2/3_Li_1/6_Fe_1/6_Co_1/6_Ni_1/6_Mn_1/3_O_2_ | 171.2 | 300 | 63.7 | ^[10]^ |
| Na_0.75_Mn_0.55_Ni_0.25_Co_0.05_Fe_0.10_Zr_0.05_O_2_ | 143 | 100 | 81 | ^[11]^ |
| Na_2_Ti_0.94_Cr_0.06_O_2_ | 217 | 50 | 83.8 | ^[12]^ |
| Na_2_Mn^3+^_0.3_Mn^4+^_2.7_O_6.85_ | 213 | 50 | 50 | ^[13]^ |
| Na_2_Ru_0.9_Mg_0.1_O_2_ | 130 | 30 | 81 | ^[14]^ |
| Na_2/3_Ni_1/3_Mn_2/3_O_2_ | 220 | 14 | 85.6 | ^[15]^ |
| Na_0.67_Cu_0.14_Mg_0.14_Mn_0.72_O_2_ | 168 | 100 | 87 | ^[16]^ |
| Na_0.67_Mn_0.75_Ni_0.25_O_2_ | 152 | 100 | 86 | ^[17]^ |
| Na_0.78_Co_1/2_Mn_1/3_Ni_1/3_O_2_ | 146 | 40 | 88 | ^[18]^ |
| Na_2/3_(Zn_0.5_Ni_0.5_)_0.3_Mn_0.7_O_2_ | 105 | 200 | 90.5 | ^[19]^ |
| Na_2/3_Cu_0.22_Mg_0.06_Mn_0.72_O_2_ | 108 | 100 | 89.7 | ^[20]^ |
| Na_0.6_Li_0.2_Mn_0.8_O_2_ | 80 | 50 | 67 | ^[21]^ |
| Na_0.5_Mg_0.15_Al_0.2_Mn_0.65_O_2_ | 177 | 100 | 56.5 | ^[22]^ |
| Na_0.67_Mg_0.2_Mn_0.8_O_2_ | 204 | 25 | 49 | ^[23]^ |
| Na_0.85_Li_0.1_Ni_0.175_Mn_0.525_Fe_0.2_O_2_ | 157 | 100 | 88 | ^[24]^ |
| NaFe_0.2_Co_0.2_Ni_0.2_Ti_0.2_Sn_0.1_Li_0.1_O_2_ | 112.7 | 100 | 81 | ^[25]^ |
| Na_0.8_Ni_0.2_Fe_0.25_Al_0.05_Mg_0.05_Ti_0.05_Mn_0.4_O_2_ | 131.9 | 200 | 80.3 | ^[26]^ |

Table S6. Transition metal dissolution concentrations in the electrolyte after 100 cycles.

|  | Li (%) | Ni (%) | Cu (%) | Mn (%) | Ti (%) |
| --- | --- | --- | --- | --- | --- |
| Na_0.8_Li_0.1_Ni_0.1_Cu_0.1_Mn_0.6_Ti_0.1_O_2_ | 0.150 | 0.013 | 0.059 | 0.029 | 0.036 |
| Na_0.67_Ni_0.33_Mn_0.67_O_2_ |  | 0.815 |  | 0.808 |  |

**References**

[1] P. Juhas, T. Davis, C. L. Farrow, S. J. L. Billinge, PDFgetX3: a rapid and highly automatable program for processing powder diffraction data into total scattering pair distribution functions, *J. Appl. Crystallogr.* **2013**, 46, 560.

[2] D. Keen, A comparison of various commonly used correlation functions for describing total scattering, *J. Appl. Crystallogr.* **2001**, 34, 172.

[3] G. Kresse, D. Joubert, From ultrasoft pseudopotentials to the projector augmented-wave method, *Phys. Rev. B* **1999**, 59, 1758.

[4] J. P. Perdew, K. Burke, M. Ernzerhof, Generalized Gradient Approximation Made Simple, *Phys. Rev. Lett.* **1996**, 77, 3865.

[5] S. Grimme, J. Antony, S. Ehrlich, H. Krieg, A consistent and accurate ab initio parametrization of density functional dispersion correction (DFT-D) for the 94 elements H-Pu, *J. Chem. Phys.* **2010**, 132, 154104.

[6] D. Wines, K. Saritas, C. Ataca, Intrinsic Ferromagnetism of Two-Dimensional (2D) MnO2 Revisited: A Many-Body Quantum Monte Carlo and DFT+U Study, *J. Phys. Chem. C* **2022**, 126, 5813.

[7] M. Nolan, Alkaline earth metal oxide nanocluster modification of rutile TiO2 (110) promotes water activation and CO2 chemisorption, *J. Mater. Chem. A* **2018**, 6, 9451.

[8] G. M. Tomboc, X. Zhang, S. Choi, D. Kim, L. Y. S. Lee, K. Lee, Stabilization, Characterization, and Electrochemical Applications of High-Entropy Oxides: Critical Assessment of Crystal Phase–Properties Relationship, *Adv. Funct. Mater.* **2022**, 32, 2205142.

[9] C. Zhao, Q. Wang, Z. Yao, J. Wang, B. Sánchez-Lengeling, F. Ding, X. Qi, Y. Lu, X. Bai, B. Li, H. Li, A. Aspuru-Guzik, X. Huang, C. Delmas, M. Wagemaker, L. Chen, Y.-S. Hu, Rational design of layered oxide materials for sodium-ion batteries, *Science* **2020**, 370, 708.

[10] L. Yao, P. Zou, C. Wang, J. Jiang, L. Ma, S. Tan, K. A. Beyer, F. Xu, E. Hu, H. L. Xin, High-Entropy and Superstructure-Stabilized Layered Oxide Cathodes for Sodium-Ion Batteries, *Adv. Energy Mater.* **2022**, 12, 2201989.

[11] Z.-Y. Li, R. Gao, L. Sun, Z. Hu, X. Liu, Zr-doped P2-Na_0.75_Mn_0.55_Ni_0.25_Co_0.05_Fe_0.10_Zr_0.05_O_2_ as high-rate performance cathode material for sodium ion batteries, *Electrochim. Acta* **2017**, 223, 92.

[12] S. Song, M. Kotobuki, Y. Chen, S. Manzhos, C. Xu, N. Hu, L. Lu, Na-rich layered Na_2_Ti_1−_*_x_*Cr*_x_*O_3−_*_x_*_/2_ (*x* = 0, 0.06): Na-ion battery cathode materials with high capacity and long cycle life, *Sci. Rep.* **2017**, 7, 373.

[13] Q. Wang, W. Yang, F. Kang, B. Li, Na_2_Mn^3+^_0.3_Mn^4+^_2.7_O_6.85_: A cathode with simultaneous cationic and anionic redox in Na-ion battery, *Energy Storage Mater.* **2018**, 14, 361.

[14] X. Li, S. Guo, F. Qiu, L. Wang, M. Ishida, H. Zhou, Na_2_Ru_1−_*_x_*Mn*_x_*O_3_ as the cathode for sodium-ion batteries, *J. Mater. Chem. A* **2019**, 7, 4395.

[15] T. Risthaus, D. Zhou, X. Cao, X. He, B. Qiu, J. Wang, L. Zhang, Z. Liu, E. Paillard, G. Schumacher, M. Winter, J. Li, A high-capacity P2 Na_2/3_Ni_1/3_Mn_2/3_O_2_ cathode material for sodium ion batteries with oxygen activity, *J. Power Sources* **2018**, 395, 16.

[16] D. Wang, F. Zou, X. Qi, S. Xu, H. Mao, D. Xiao, S. Lu, B. Guo, Y. Lyu, Local Structure Regulation for Oxygen Redox and Structure Stability of P2-Type Cathodes, *Small* **2025**, 21, 2411052.

[17] W. Kong, R. Gao, Q. Li, W. Yang, J. Yang, L. Sun, X. Liu, Simultaneously tuning cationic and anionic redox in a P2-Na_0.67_Mn_0.75_Ni_0.25_O_2_ cathode material through synergic Cu/Mg co-doping, *J. Mater. Chem. A* **2019**, 7, 9099.

[18] C. Hakim, N. Sabi, L. A. Ma, M. Dahbi, D. Brandell, K. Edström, L. C. Duda, I. Saadoune, R. Younesi, Understanding the redox process upon electrochemical cycling of the P2-Na_0.78_Co_1/2_Mn_1/3_Ni_1/6_O_2_ electrode material for sodium-ion batteries, *Commun. Chem.* **2020**, 3, 9.

[19] A. Konarov, H. J. Kim, J.-H. Jo, N. Voronina, Y. Lee, Z. Bakenov, J. Kim, S.-T. Myung, High-Voltage Oxygen-Redox-Based Cathode for Rechargeable Sodium-Ion Batteries, *Adv. Energy Mater.* **2020**, 10, 2001111.

[20] P.-F. Wang, Y. Xiao, N. Piao, Q.-C. Wang, X. Ji, T. Jin, Y.-J. Guo, S. Liu, T. Deng, C. Cui, L. Chen, Y.-G. Guo, X.-Q. Yang, C. Wang, Both cationic and anionic redox chemistry in a P2-type sodium layered oxide, *Nano Energy* **2020**, 69, 104474.

[21] X. Rong, J. Liu, E. Hu, Y. Liu, Y. Wang, J. Wu, X. Yu, K. Page, Y.-S. Hu, W. Yang, H. Li, X.-Q. Yang, L. Chen, X. Huang, Structure-Induced Reversible Anionic Redox Activity in Na Layered Oxide Cathode, *Joule* **2018**, 2, 125.

[22] M. Jia, H. Li, Y. Qiao, L. Wang, X. Cao, J. Cabana, H. Zhou, Elucidating Anionic Redox Chemistry in P3 Layered Cathode for Na-Ion Batteries, *ACS Appl. Mater. Interfaces* **2020**, 12, 38249.

[23] E. J. Kim, L. A. Ma, D. M. Pickup, A. V. Chadwick, R. Younesi, P. Maughan, J. T. S. Irvine, A. R. Armstrong, Vacancy-Enhanced Oxygen Redox Reversibility in P3-Type Magnesium-Doped Sodium Manganese Oxide Na_0.67_Mg_0.2_Mn_0.8_O_2_, *ACS Appl. Energy Mater.* **2020**, 3, 10423.

[24] Y. You, S. Xin, H. Y. Asl, W. Li, P.-F. Wang, Y.-G. Guo, A. Manthiram, Insights into the Improved High-Voltage Performance of Li-Incorporated Layered Oxide Cathodes for Sodium-Ion Batteries, *Chem* **2018**, 4, 2124.

[25] K. Tian, H. He, X. Li, D. Wang, Z. Wang, R. Zheng, H. Sun, Y. Liu, Q. Wang, Boosting electrochemical reaction and suppressing phase transition with a high-entropy O3-type layered oxide for sodium-ion batteries, *J. Mater. Chem. A* **2022**, 10, 14943.

[26] L. Yu, X. Ma, L. Yang, Q. Guo, S. Ye, N. Ahmad, Z. Jiang, J. Liang, J. Xia, B. Peng, W. He, L. Shi, G. Zhang, Suppressing the high-voltage phase transition in O3-type layered cathode enables ultra-stable sodium-ion batteries, *Energy Storage Mater.* **2025**, 83, 104679.
